# Strengthening digital competencies in India’s health workforce: development and feasibility evaluation of a digital health competency framework for frontline healthcare workers in Uttar Pradesh

**DOI:** 10.1093/oodh/oqag009

**Published:** 2026-05-08

**Authors:** Chirag Bariya, Himanshu Burad, Rukmani Keshav, Surbhi Arul, Umesh Jhadav, Padmini Vishwanath, Girish Patil, Karthik Adapa

**Affiliations:** International Innovation Corps, University of Chicago Trust, Ground Floor, DLF Capitol Point Building, Baba Kharak Singh Marg, Near Hanuman Temple, Connaught Place, New Delhi, Delhi 110001, India; International Innovation Corps, University of Chicago Trust, Ground Floor, DLF Capitol Point Building, Baba Kharak Singh Marg, Near Hanuman Temple, Connaught Place, New Delhi, Delhi 110001, India; International Innovation Corps, University of Chicago Trust, Ground Floor, DLF Capitol Point Building, Baba Kharak Singh Marg, Near Hanuman Temple, Connaught Place, New Delhi, Delhi 110001, India; International Innovation Corps, University of Chicago Trust, Ground Floor, DLF Capitol Point Building, Baba Kharak Singh Marg, Near Hanuman Temple, Connaught Place, New Delhi, Delhi 110001, India; International Innovation Corps, University of Chicago Trust, Ground Floor, DLF Capitol Point Building, Baba Kharak Singh Marg, Near Hanuman Temple, Connaught Place, New Delhi, Delhi 110001, India; Koita-Centre for Digital Health, Ashoka University, Plot No. 2, Rajiv Gandhi Education City, National Capital Region P.O. Rai, Sonepat, Haryana 131029, India; Koita-Centre for Digital Health, Ashoka University, Plot No. 2, Rajiv Gandhi Education City, National Capital Region P.O. Rai, Sonepat, Haryana 131029, India; Department of Health Systems, World Health Organization—South-East Asia Regional Office, World Health House, Indraprastha Estate, Mahatma Gandhi Marg, New Delhi, Delhi 110002, India

**Keywords:** digital health competency framework, frontline workers, evaluation, feasibility, low- and middle-income countries, workforce preparedness

## Abstract

The rapid growth of digital health initiatives has heightened reliance on frontline health workers (FLHWs) to deliver, document, and manage services through digital tools, particularly in low- and middleincome countries (LMICs). In India, the widespread rollout of platforms under the Ayushman Bharat Digital Mission (ABDM) is not yet matched by a standardized digital health competency framework (DHCF) for FLHWs, hindering systematic skill development, assessment, and integration. This study designed, developed, and evaluated a theory-driven, evidence-based, and scalable DHCF for India's health workforce. Framed as a feasibility and proof-ofconcept study, it was piloted among FLHWs in Uttar Pradesh using a three-stage approach comprising design, implementation, and evaluation. The framework development drew on a systematic literature review and the Government of India's Framework for Roles, Activities, and Competencies (FRAC). A cadre-agnostic competency dictionary was created, spanning functional, behavioral, domainspecific, and intervention-specific skills across graded proficiency levels. Competencies were mapped to FLHW roles, and aligned training materials and assessments were developed. The framework was piloted through in-person, instructor-led sessions for Auxiliary Nurse Midwives (ANMs) in two districts (*n* = 70), alongside baseline assessments for Accredited Social Health Activists (ASHAs; *n* = 32). The resulting DHCF comprises a three-component package: (i) a cadre-agnostic competency dictionary with progressive proficiency levels; (ii) systematic role-tocompetency mapping using the FRAC methodology; and (iii) integrated training content and assessment scaffolding designed for institutional embedding. The framework defined 10 core competencies, enabling role-specific mapping across cadres. Feasibility testing demonstrated significant gains in ANMs' knowledge and digital skills: Wilcoxon signed-rank tests showed significant improvements in two of four competency levels (C1L1 and C2L1; both *P* < .001), with the largest effect for data collection basics (*r* = 0.84). ASHA baseline assessments revealed substantial foundational literacy gaps (mean total score 11.97/30 [39.9%]; data collection was the weakest competency at 32.5%, with no ASHA scoring above 60% on C2L1). Stakeholders affirmed the framework's relevance, feasibility, and adaptability, while identifying the need for hybrid training models and stronger institutional embedding.The DHCF offers a structured, scalable approach to standardizing digital health training for FLHWs and strengthening workforce preparedness in resource-limited settings during India's digital health transition. This feasibility study establishes the framework's relevance and applicability; future work is needed to evaluate effectiveness at scale, long-term competency retention, and linkage to service delivery outcomes. Parallel attention to digital tool design and usability will be essential to complement competency-building efforts.

## Introduction

Digital health has become an increasingly integral component of health systems globally, with countries rapidly adopting digital health technologies to accelerate progress towards universal health coverage (UHC) (Sylla et al. 2025). The World Health Organization (WHO) defines digital health as the field of knowledge and practice associated with the development and use of digital technologies to improve health (World Health Organization 2024). This broad definition encompasses a wide range of eHealth solutions, smart devices, and emerging technologies, including the Internet of Things, big data analytics, artificial intelligence, and robotics. Collectively, these technologies have been leveraged to strengthen health systems in multiple ways, including expanding access to care through telemedicine and mobile health platforms (Agarwal et al. 2020; Maroju et al. 2023), supporting clinical decision-making (Grechuta et al. 2024), improving patient information management through electronic health records (Kirwa et al. 2025), and enabling real-time disease surveillance and monitoring through data-driven analytics (Schulz et al. 2020; Shausan et al. 2023).

India has made substantial progress in institutionalizing digital health within its healthcare system. The National Health Policy (2017) formally recognized digital health as a key enabler of UHC, paving the way for the launch of the ABDM in 2020 (Ministry of Health and Family Welfare, Government of India 2017). ABDM seeks to establish a unified, interoperable digital health ecosystem nationwide (Arjun et al. 2024; Narayan et al. 2024). Over the past 5 years, digital health interventions have been increasingly deployed to address public health challenges, including large-scale vaccination management and monitoring through platforms such as CoWIN (Rana et al. 2024; Vasanthan et al. 2024), as well as last-mile telemedicine delivery through initiatives such as eSanjeevani (Naithani et al. 2021).

Within this national context, Uttar Pradesh (UP), India’s most populous and resource-constrained state, home to ~16.5% of the country’s population (Anjum and Singh 2024; World Bank 2016), presents a compelling setting for examining digital health implementation. With 77.7% of its population residing in rural areas (Bhardwaz and Kumar 2023) and the state ranking lowest nationally in overall health performance (NITI Aayog 2021), UP faces persistent challenges in ensuring equitable and timely health service delivery. In response, the Government of Uttar Pradesh (GoUP) has initiated several digital health measures, including the development of an integrated COVID-19 mobile platform to support testing, treatment, and longitudinal care (Srivastava and Sonar 2023), as well as the integration of UP-HMIS to address gaps in the national Health Management Information System (Meghani et al. 2022).

Frontline health workers (FLHWs)—including Auxiliary Nurse Midwives (ANMs), Accredited Social Health Activists (ASHAs), and Anganwadi workers (AWWs)—are central to the effective deployment of these digital health initiatives. These cadres constitute the backbone of India’s primary healthcare system (Kalne and Mehendale 2022). In Uttar Pradesh alone, more than 160 000 ASHA workers serve a predominantly rural population of ~232 million, making them the largest and most heavily burdened FLHW cadre nationally (National Health Systems Resource Centre 2026). As the first point of contact for populations in remote and underserved communities (Dhaliwal et al. 2025), FLHWs play a critical role in bridging communities and the formal health system. Through the delivery of essential interventions—such as antenatal care, institutional delivery support, immunization services, public health surveillance, and health education—ASHAs, ANMs, and AWWs substantially contribute to improved health outcomes in UP and across India (Kalne and Mehendale 2022; Sankar et al. 2024).

The digitalization of frontline service delivery has significantly expanded the scope and complexity of FLHWs’ day-to-day responsibilities. FLHWs increasingly operate across multiple digital platforms to collect patient data, monitor service delivery, and report health indicators in real time, often under challenging field conditions (Longhini et al. 2022). Evidence from a multi-state study in India indicates that ASHA workers routinely use several applications, including NCD-GOI ANM, ASHA Sarvekshan, and Divyang Sarthi, to fulfill their reporting and service delivery functions (Sreerupa 2024). In 2021, GoUP further piloted e-Kavach, a comprehensive digital job aid enabling longitudinal patient tracking through unique identifiers such as the Ayushman Bharat Health Account (ABHA) (India Health Action Trust 2025). As digital workloads expand, equipping FLHWs with appropriate training and competencies has become essential. Inadequate digital proficiency risks reduced efficiency, compromised data quality, and delays in service delivery, ultimately affecting care quality for vulnerable populations (Longhini et al. 2022; Wamala Andersson and Gonzalez 2025).

Digital literacy—defined as the ability to understand and use information from multiple sources and formats via digital technologies (Knobel and Lankshear 2006)—is foundational to effective participation in an increasingly digitalized health system. By enabling FLHWs to access information, manage services, and support informed clinical and programmatic decision-making (Longhini et al. 2022; Borges do Nascimento et al. 2023; Wamala Andersson and Gonzalez 2025), digital literacy underpins the successful implementation of digital health interventions. Despite its importance, persistent challenges impede digital capacity building among FLHWs. The existing literature points to fragmented and non-standardized training approaches (Gudi et al. 2021; Newton-Lewis and Nanda 2021; Singh et al. 2021) and workforce burnout (UNICEF 2025) as major barriers. Compounding these challenges, national initiatives such as ABDM currently lack standardized guidelines specifying the digital competencies required of FLHWs (Samudyatha et al. 2023; Sharma et al. 2023). This absence of standardization hampers both effective training design and systematic assessment of competency acquisition, particularly in high-burden states such as Uttar Pradesh.

One evidence-based approach to addressing these challenges is the use of a Digital Health Competency Framework (DHCF) (Car et al. 2025; Framework|Workforce, Training and Education | NHS England 2019). A DHCF defines a structured set of knowledge, skills, and abilities required to strengthen digital health capacity across health systems, encompassing health professionals, program managers, policymakers, and communities (World Health Organization 2023). Several DHCFs have been developed in high-resource settings, including the DECODE framework for medical education (Car et al. 2025), the National Health Service (NHS) digital workforce framework in the UK (Framework | Workforce, Training and Education | NHS England 2019), and frameworks developed by the Australian Digital Health Agency (Australian Digital Health Agency 2020). Additional examples include the Health Information Technology Competencies framework, the TIGER framework, and standards developed by the Australian Nursing and Midwifery Federation (ANMF) (Australian Digital Health Agency 2020; Hübner et al. 2019; Nazeha et al. 2020; Longhini et al. 2022).

While these frameworks provide valuable guidance for building digital capacity among healthcare professionals, they predominantly focus on physicians, clinicians, and managerial staff (Nazeha et al. 2020; Longhini et al. 2022). To the best of our knowledge, there remains no DHCF specifically tailored to the needs, contexts, and constraints of FLHWs operating in low-resource settings (Rana et al. 2024; Bhandari et al. 2025).

In response to this gap, we conceptualized the DHCF as an integrated three-component package: (i) a cadre-agnostic competency dictionary with progressive proficiency levels, (ii) a systematic role-to-competency mapping process using the Framework for Roles, Activities, and Competencies (FRAC) methodology, and (iii) training content and assessment scaffolding designed for institutional embedding through existing government mechanisms. Unlike prior frameworks that define competencies at a high level, this package anchors competencies in the day-to-day tasks of FLHWs, linking digital skills directly to routine service delivery workflows and enabling structured, measurable capacity building.

## Objective

In response to the growing digital responsibilities of FLHWs and the absence of a standardized DHCF within national policy, this study aimed to design, develop, and evaluate a theory-driven, evidence-informed, and scalable DHCF for India’s health workforce. The framework was piloted and assessed as a feasibility and proof-of-concept study among FLHWs in Uttar Pradesh to assess its applicability, feasibility, and relevance at the grassroots level.

## Methodology

**Figure 1:**
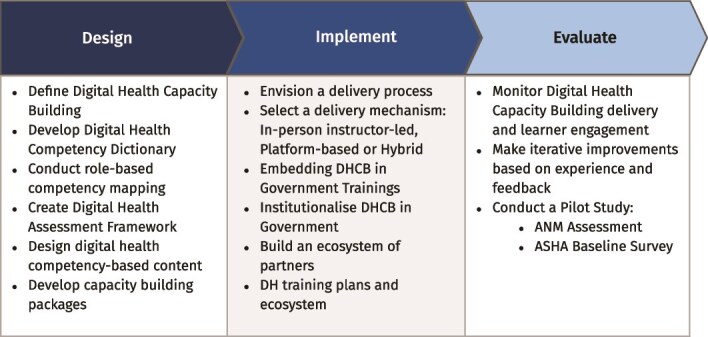
Flow diagram of the three-stage development process.

The development of the DHCF for India’s health workforce followed a three-stage process (Fig. 1): (i) Design, (ii) Implement, and (iii) Evaluate. This section outlines the key steps undertaken across these stages. While the framework was conceptualized for the broader health workforce, its initial application and evaluation were conducted with FLHWs in Uttar Pradesh, serving as a feasibility and proof of concept for scalability and contextual adaptation across other health cadres.

### Design

The DHCF was developed through a structured design process involving several meticulous steps. This section describes the specific steps that were taken to design the DHCF.

#### Step 1: Literature review

To ground our design in evidence, an extensive literature review of existing digital health capacity-building initiatives and frameworks worldwide was conducted. Key search terms used in the literature review were “Digital health capacity building,” “Digital health competencies,” “Digital Health Frameworks,” “Frameworks for capacity building in healthcare”; “m-health competencies,” “Digital health literacy,” “Digital tools for training FLHWs.” These search terms were used to review global scholarly papers, frameworks, strategies, and policies related to digital health capacity building (DHCB) for health workers. Databases searched included PubMed, Scopus, and Google Scholar. Respective websites of organizations were also accessed to retrieve frameworks (NHS, State of Victoria, Department of Health 2022; Victoria, WHO, DECODE, and C-LOP).

#### Step 2: Defining Digital Health Capacity Building

A definition of DHCB was developed by examining the integration of three core components: Health, Capacity Building, and Digital Health. Definitions that were used for Health and Digital Health, and Capacity Building are as follows: Health, as defined by the WHO, is “a state of complete physical, mental, and social well-being and not merely the absence of disease or infirmity” (Svalastog et al. 2017). Digital Health, as defined by the WHO, field of knowledge and practice associated with the development and use of digital technologies to improve health (WHO 2019). Capacity Building, as defined by the UN, is the process of developing and strengthening the skills, instincts, abilities, processes, and resources that organizations and communities need to survive, adapt, and thrive in a fast-changing world (United Nations 2023).

#### Step 3: Identification of a standardized approach

Six frameworks identified in Step 1 were examined and considered for adaptation. A thorough review was conducted to select the framework most appropriate for the Indian context. Factors taken into account included relevance and applicability to India’s health system architecture, familiarity of government officials with the framework, adaptability to diverse cadre roles, and appropriateness for FLHWs operating in resource-constrained settings. Beyond these academic and operational criteria, the selection was intentionally calibrated to align with the Government of India’s broader strategic shift under Mission Karmayogi. India’s national training policy is moving decisively from a “Rule-to-Role” philosophy—transitioning from program-based information delivery to a role-centric, competency-driven model of workforce development. In designing the DHCF, we sought to ground the framework in the core principles of the FRAC, the methodology underpinning Mission Karmayogi’s competency architecture. By anchoring the DHCF in these Government of India-mandated pillars, the study ensures that the resulting competencies for FLHWs are not only theoretically sound and evidence-informed but also technically compatible with official government training programs and the iGOT-Karmayogi digital learning ecosystem. This alignment is a deliberate design choice: it positions the DHCF to leverage existing institutional infrastructure for scale-up, while contributing evidence to support the broader transition from program-based training to role-centric capacity building through a competency dictionary approach.

#### Step 4: Defining the Digital Health landscape

Given the vast scope of the term ‘Digital Health’ and India’s rapid technological advancements, we sought to narrow the focus of our study. To define our Digital Health landscape, we drew upon systems across three levels of digital health—global, national, and state. The global level helped us define the overarching framework, the national level outlined the landscape within India, and the state level provided the local context of Uttar Pradesh. At the global level, the NHS (UK) DHCF was selected as a reference model, being among the first internationally developed frameworks. At the national level, the ABDM was identified due to its clear role in providing governance, standardization, and a unified digital health infrastructure for India. At the state level, the Uttar Pradesh Digital Health Roadmap was chosen as it outlines the state-specific priorities and strategies.

#### Step 5 & 6: Identifying competencies, developing digital health competency dictionary & mapping competencies

**Figure 2:**
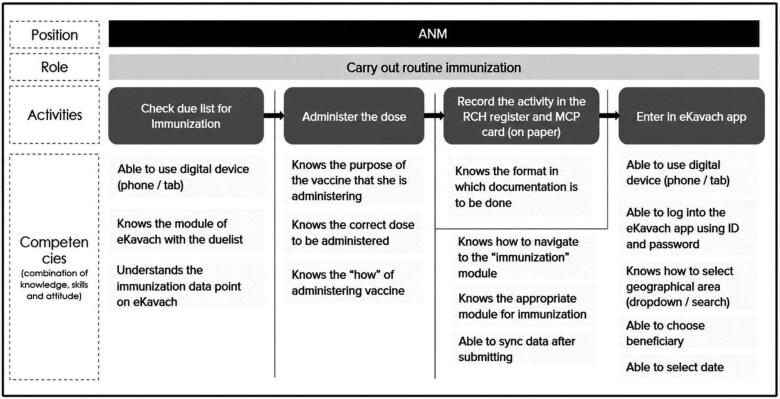
Example of the FRAC-ing process.

The selected standardized approach—the FRAC method, was followed to identify the digital health competencies. An example of the FRAC method’s application to ANMs is shown in Fig. 2. We began by identifying the key components of our Digital Health landscape. For each component, we mapped an exhaustive list of roles and activities across four different levels of engagement—community, facility, district, and state. The ABHA was one such component. For this component, at the community and facility levels, the envisioned roles included enumerating ABHAs and generating digital health records linked to ABHAs. At the district and state levels, the focus shifted to the analysis of ABHA-linked health records to enable evidence-based decision-making. Based on these roles and activities for the ABHA component, we identified specific digital health competencies required for their effective implementation. At the community and facility levels, these included data collection using digital tools and ensuring data privacy. At the district and state levels, competencies expanded to include data analysis, data privacy, and data management. Finally, these competencies were validated against international frameworks and existing global literature.

The competency identification process involved 12 stakeholder consultation sessions conducted over a period of 3 months, including iterative rounds of mapping and validation (3 rounds). Each round involved cross-referencing identified competencies with international frameworks (NHS, ANMF, DECODE) and field observations from FLHW settings. The process required ~480 person-hours of technical work across the research team.

A digital health competency dictionary, a cadre-agnostic tool, is a dynamic repository of competencies required by all cadres and roles. A competency dictionary typically provides a detailed catalog of essential digital competencies categorized across individual, institutional, and systemic levels for varied healthcare cadres. Since digital health is an evolving space, the dictionary was designed to be agile and cadre-agnostic. Based on the digital health competencies identified and drawing inspiration from the competency levels outlined in the Mission Karmayogi Framework (Department of Personnel and Training, Government of India 2020), a Digital Health Competency Dictionary was developed. This dictionary clearly defines each competency and further breaks it down into measurable sub-components. The competencies were classified into four categories, three of which were adapted from Mission Karmayogi. The three competencies from Mission Karmayogi included functional, behavioral, and domain competencies, while the fourth, digital intervention-specific competencies, was developed to meet the unique needs of individual interventions. The definitions of each group of competencies are as follows:

- **Functional digital competencies:** knowledge, skills, and abilities required to operate in a digital environment across various functions or positions.
- **Behavioral digital competencies:** motives, skills, and traits that enable individuals to promote and lead digital initiatives.
- **Digital health specific domain competencies:** specialized knowledge, skills, and abilities specific to the digital health domain, applicable to specific organizations or functions.
- **Digital intervention-specific competencies:** competencies tailored to specific digital interventions, focusing on their modules and functionalities.

This dictionary was developed as an evolving and flexible tool to be adapted as per the requirements of the training program and the target audience. The five proficiency levels—Basic, Proficient, Advanced, Expert, and Master (Levels 1 through 5, respectively; see Table 1)—were adapted from the cognitive tiers of Bloom’s taxonomy, ensuring that the depth and complexity of training content are calibrated to the functional demands of each role.

**Table 1:** Participant flow by assessment component.

Assessment component	Barabanki	Sitapur	Total	Paired *n*
Enrolled	32	38	70	—
Baseline C1L1	26	34	60	—
Endline C1L1	25	32	57	—
Paired C1L1 (baseline + endline)	26^a^	32	58	58
Baseline C1L2	24	35	59	—
Endline C1L2	24	28	52	—
Paired C1L2	29^b^	28	57	57
Baseline C2L1	17	30	47	—
Endline C2L1	18	25	43	—
Paired C2L1	16	24	40	40
Baseline C2L2	18	30	48	—
Endline C2L2	19	23	42	—
Paired C2L2	18	23	41	41
Skill demo C1 (L1 + L2)	18	18	36	—
Skill demo C2L1	17	18	35	—

^a^Some ANMs had endline without baseline for C1L1 due to late joining; paired n reflects those with both.

^b^Discrepancy reflects combined session counts across weeks.

#### Step 7: Development of the Digital Health Assessment Framework

Once the competencies and the dictionary were finalized, a Digital Health Assessment Framework was developed to offer a standardized and structured approach to evaluate the acquisition of these competencies after a capacity-building training session. Test items were designed based on the nature and level of the competencies being assessed. Every assessment instrument was categorized into one of the three domains—Knowledge, Skill, and Attitude. Knowledge includes a respondent’s awareness and understanding of theories, concepts, and processes. Skill refers to the know-how and practice of knowledge, such as the ability to communicate via or use a digital device. Attitude includes respondents’ motivation and value judgments regarding tasks, such as the perceived usefulness of a digital device or self-efficacy in carrying out a task on a digital device. At every competency level, it was deemed essential to contain assessments testing all three domains (knowledge, skills, and attitudes).

Knowledge-based competencies were mapped to multiple-choice questions (MCQs), fill-in-the-blank items, and open-ended essay responses. Skill-based competencies were linked with case studies and direct observations, while attitude-based competencies were to be assessed via direct observations and interviews. This approach ensured alignment between intended learning outcomes and actual learner performance. Importantly, the question bank was designed to be flexible, allowing for regular updates and revisions to maintain contextual and content relevance.

For the pilot assessments, 40 MCQs were selected for the ANM pilot (10 per competency-level: C1L1, C1L2, C2L1, C2L2) and 30 MCQs for the ASHA baseline (10 each for C1L1, C1L2, C2L1), drawn from the full bank of 250 MCQs. The selection blueprint ensuring coverage across all competency levels is provided in Supplementary Table S2. Four reviewers (two digital health subject-matter experts, one assessment design specialist, and one public health professional with FLHW training experience) independently evaluated each MCQ for: (i) accurate mapping to relevant competency codes, (ii) grammatical correctness of the question stem, (iii) clarity and mutual exclusiveness of answer alternatives, (iv) consistency in the length and structure of options, and (v) logical ordering of response choices. Disagreements were resolved through consensus discussion. Of 250 MCQs in the full bank, 218 (87.2%) were retained after review, 22 (8.8%) were revised (re-mapped to correct competency codes or reworded for clarity), and 10 (4.0%) were dropped. The term “content review and face validity” is used to describe this validation process, as formal psychometric validation was not conducted in this phase.

The representative MCQ items (3 sample items per competency assessed in the pilot) are provided in Supplementary Material 2 (Table S2).

#### Step 8: Review and feedback

The Framework was reviewed and vetted for relevance, content review, face validity, reliability, and comprehensiveness by two groups: (i) key domain experts and senior public-health professionals with technical expertise from the Uttar Pradesh Technical Support Unit (UPTSU); and (ii) frontline ANMs.

ANMs were engaged in two stages—pre-training and post-training. First, pre-training consultations were conducted across selected Community Health Centers (CHCs) and Primary Health Centers (PHCs) with 17 ANMs from CHCs in Gonda, 19 ANMs from CHCs in Ayodhya, and 5 ANMs from a PHC in Kursi, Barabanki. Then, the pilot phase engaged 70 ANMs from Barabanki and Sitapur. ANM feedback was gathered on the following dimensions: (i) Competency Relevance through facilitator’s observation (“Is this competency related to any activities you do as an ANM? If yes, which?”, (ii) Need for training, (iii) Prior Training Coverage, (iv) Exhaustiveness (“Are there any relevant competencies not included?”). and (v) Open-ended feedback on the training (“How can we make this competency better?”). Post-training, ANMs were consulted for their experiential reflections on applicability and learning. Collecting feedback at both stages ensured that the framework incorporated both initial expectations from the field and practical insights following training.

#### Step 9: Creating competency-mapped content

First, relevant content for the mapped competencies was identified and outlined. To develop this content outline, we consulted several sources, including the - Objective Structured Clinical Examination (OSCE) Framework assessment method in healthcare education (Afzal et al. 2023), the Training Manual on Designing Proctored Independent Authorized Assessments (Centre for Effective Governance of Indian States), the National Digital Health Blueprint (NHA 2020), and the 4E3P digital professionalism model (Mather and Cummings 2019). In addition, the team reviewed digital diagnostic tools used in the NHS to introduce staff to digital capabilities (Newman et al. 2019). Based on these resources, a course blueprint was developed that mapped learning outcomes to each competency level, which in turn guided the creation of instructional content and corresponding assessment tools for each level. This blueprint informed the design of structured training modules—referred to as the DHCB Packages—ensuring that all learning outcomes defined under each competency were systematically addressed through aligned content and evaluation instruments. The next step was to align instructional methods and learning materials, such as videos, Animations, Diagrams, Flowcharts, Infographics and Simulations, with the needs of each module. Once this alignment was done, the most appropriate mode of learning, including tools and technologies for engagement and learning, was determined based on whether the course was to be conducted in-person, virtually, or based on learning management systems. In the final stage, the learning content was cross-checked with the assessment items to guarantee that all competencies being evaluated were effectively integrated into the instructional material.

### Implementation

After the design phase was completed, a blueprint for implementation was developed. The blueprint for implementation included three components: (i) delivery process, (ii) delivery mechanisms, and (iii) process for embedding digital health capacity-building (DHCB) packages in government trainings.

#### Delivery process

The team proposed a step-by-step delivery process to be followed by stakeholders interested in leveraging the DHCF to deliver capacity-building training programs. The first step involves (i) aligning the specific competencies with the cadre in question for a particular training. Once the competencies are identified, (ii) a training calendar is to be developed to schedule training sessions. This is to be followed by a (iii) pre-assessment, where health workers are evaluated on their existing knowledge and digital skills. Based on the pre-assessment, eligible individuals are to be nominated for training. These nominees then (iv) undergo capacity building through appropriate content and methodology. After training, a (v) post-assessment is conducted to evaluate knowledge gained and competencies acquired. Candidates then move to the (vi) certification stage. To ensure sustainability, competency is integrated into performance reviews, and based on performance and evolving needs, health workers are enrolled in a (vii) refresher training.

#### Delivery mechanism

The delivery mechanism was designed to integrate the developed training content into existing digital platforms and systems and to be adaptable to both in-person and hybrid modalities. For in-person, instructor-led training, we proposed embedding DHCB modules within ongoing digital intervention-based training programs. These sessions could be delivered through existing forums such as cluster meetings among FLHWs or other routine training platforms. For the hybrid approach, we recommended incorporating DHCB modules into government-led induction programs for ANMs and Skilled Birth Attendants, as well as into digital training initiatives conducted by ABDM and the State Digital Health Mission, which are delivered online.

#### Institutionalization of the digital health competency framework

For sustained impact and integration into existing health systems, the team developed the DHCF with the aim for it to be adaptable to multiple administrative levels. At the state level, we proposed that DHCB reviews be conducted under the leadership of the Additional Chief Secretary of the Department of Medical Health, with the involvement of divisional-level stakeholders. At the district level, we recommended that review meetings be led by the Chief Medical Officer (CMO) and that the District Health Society provide opportunities to assess progress. At the block and facility levels, we proposed that the components of the DHCF be systematically integrated into routine review agendas, systematic mentoring programs, and facility competency passbooks. Moreover, for frontline healthcare workers, cluster meetings could serve as an effective platform for monitoring and facilitating progress in DHCB initiatives.

### Evaluation

To assess the progress of key beneficiaries in acquiring digital health competencies, a structured approach was proposed to monitor learner engagement and gather feedback. In addition, feasibility pilot studies and baseline assessments were conducted to evaluate both the DHCB packages and the completeness of the assessment framework.

#### Monitoring learner engagement and feedback

The proposed approach for monitoring learner engagement and feedback was designed as a three-step process to ensure continuous learning from data and feedback. The first proposed step was to systematically collect data on learner engagement, with tailored approaches for in-person and learning management system (LMS)-based capacity-building programs. For in-person, instructor-led training, data collection is to be centered on gathering learner feedback and conducting pre- and post-assessments. For platform-based programs, the approach includes capturing data from multiple sources, such as user sign-ups, login activity, time spent on modules, pre- and post-assessment scores, and the number of competency certificates earned. The next step proposed was to review and analyze the data acquired. Two methods to do this would be to conduct (i) program monitoring on key metrics such as attendance and completion, and (ii) analysis, including examining engagement by factors such as cadre, geographic location, and course type, to identify any gaps or challenges. The third step is to incorporate the lessons from the data review and analysis into program improvement.

#### Pilot study with auxiliary nurse midwives

**Figure 3:**
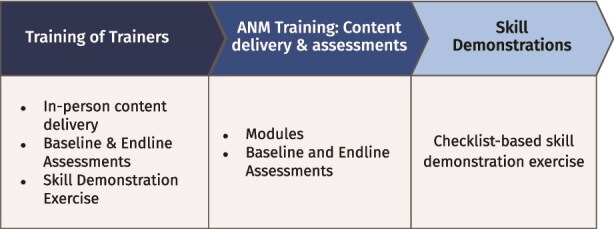
Flow of the implementation plan of the ANM pilot.

A feasibility pilot study was designed and conducted with the objective of operationalizing and evaluating the framework for FLHWs through a structured training program (Fig. 3). Two districts were identified for the pilot—Barabanki and Sitapur, selected for their representativeness of UP’s rural healthcare landscape and the availability of existing training infrastructure at CHCs. The study was conducted during weekly ANM meetings at CHCs, enabling integration with routine health system activities.

##### Participants

A total of 70 ANMs were enrolled (32 from Barabanki, 38 from Sitapur). Participants were ANMs serving at sub-centers and PHCs under the selected CHCs who attended the weekly ANM meeting during the study period. No formal exclusion criteria were applied; all ANMs present during training sessions were invited to participate. Demographic data were collected for a subset of participants: in Barabanki, 32 ANMs provided age data (mean age: 36.7 years, range: 23–58 years), with educational qualifications including Intermediate (most common), High School, BA, MA, and B.Sc. In Sitapur, limited demographic data were available for 11 ANMs (mean age: 34.9 years, range: 25–58 years).

##### Training intervention

Training was delivered through 1-h in-person, instructor-led sessions during weekly ANM meetings at CHCs, conducted over 2 weeks per district (four sessions total per district). Sessions were facilitated by master trainers (qualified staff nurses and nurse mentors), with Block Program Managers and Block Community Process Managers as substitute trainers. The training covered two functional digital competencies: C1—Using digital devices and applications, and C2—Data collection and Management using digital devices. Training content was delivered as competency-mapped DHCB packages, incorporating visual aids, device-based practice, and scenario-based learning activities.

##### Assessment instruments

*Knowledge assessments*

Knowledge was assessed via pen-and-paper MCQs at baseline (pre-training) and endline (post-training) for each competency level. Each competency level was assessed using 10 MCQs, yielding a maximum score of 10 per competency level. Some items had a single correct response, while others had multiple correct options; for multiple-response items, the answer was marked correct only if all correct options were selected. No partial marks were awarded. Assessments were administered for all four competency levels (C1L1, C1L2, C2L1, C2L2), giving a total of 40 MCQs per complete assessment cycle. Not all ANMs completed assessments for all competency levels due to scheduling constraints during weekly meetings, resulting in variable sample sizes across competency levels. Baseline and endline MCQ sets used matched but not identical items (Set A for baseline, Set B for endline), with questions mapped to the same competency codes and difficulty levels.

*Skill demonstration assessments*

Practical competencies were assessed through a structured skill demonstration exercise using a standardized observation checklist with binary (Yes = 1/No = 0) scoring. The C1 demonstration (Levels 1 and 2 combined) comprised nine tasks (maximum score: 9), covering device operations such as switching on a smartphone, unlocking the screen, connecting to the internet, installing and uninstalling an application, and joining an online meeting. The C2L1 demonstration comprised 14 tasks (maximum score: 14), covering data collection operations including navigating to the appropriate application module, entering patient data, selecting beneficiaries, and syncing data. Skill demonstrations were scored by trained master trainers. Inter-rater reliability was not formally assessed; however, the binary scoring format was selected to minimize assessor subjectivity.

Training was delivered over four sessions across 2 weeks in each district. Of the 70 enrolled, not all ANMs completed every assessment due to scheduling constraints. The number of ANMs with paired baseline-endline data varied by competency level: C1L1 (*n* = 58), C1L2 (*n* = 57), C2L1 (*n* = 40), and C2L2 (*n* = 41). Skill demonstrations were completed by 36 ANMs for C1 (L1 + L2) and 35 for C2L1(18 per district for C1; 17 in Barabanki and 18 in Sitapur for C2L1); the remaining ANMs were unavailable for skill demonstration sessions due to scheduling conflicts with clinical responsibilities. Pre- and post-assessments were paired within individuals using unique participant codes.

##### Participant flow and missing data

Of the 70 ANMs enrolled, not all completed every assessment due to scheduling constraints inherent to the weekly ANM meeting format and competing clinical responsibilities. The participant flow by assessment component is shown in Table 1.

Pre/post assessments were linked within individuals using unique serial numbers assigned at enrollment. Only ANMs with both baseline and endline scores for a given competency level were included in paired pre-post comparisons.

##### Statistical analysis

Descriptive statistics were computed for each competency level at baseline and endline, including means, standard deviations (SDs), medians, and interquartile ranges (IQRs). Score distributions were assessed for normality using the Shapiro–Wilk test. Given that the majority of score difference distributions deviated significantly from normality (Shapiro–Wilk *P* < .05 for C1L1, C1L2, and C2L2), non-parametric methods were used as the primary analytic approach across all competency levels for consistency. The Wilcoxon signed-rank test was used for paired pre-post comparisons. As a sensitivity analysis, paired t-tests were also conducted. Effect sizes were computed as both rank-biserial correlation (*r*) for the Wilcoxon test and Cohen’s d for the paired differences. Effect sizes were interpreted as small (*r* = 0.1–0.3, d = 0.2–0.5), medium (*r* = 0.3–0.5, d = 0.5–0.8), and large (*r* > 0.5, d > 0.8). Threshold-based analyses were also conducted, reporting the percentage of ANMs scoring ≥80% (≥8/10) and ≥90% (≥9/10) at baseline and endline. For skill demonstrations, the percentage of ANMs scoring ≥90% was computed against the respective maximum scores (9 for C1, 14 for C2L1). All analyses were conducted in Python (v3.12) using SciPy (v1.11) and NumPy (v1.26). Statistical significance was set at α = 0.05.

#### Baseline assessment with accredited social health activists

A baseline assessment was conducted with 32 ASHAs in the Barabanki district at the CHC Satrikh facility. All ASHAs assigned to the CHC Satrikh catchment area who presented at the facility on the assessment day were invited to participate; no formal inclusion or exclusion criteria were applied. The sample included ASHAs of varied age groups and literacy levels, reflecting the heterogeneity typical of this cadre in rural Uttar Pradesh.

A pen-and-paper-based assessment was used to evaluate the ASHAs’ foundational knowledge and understanding of digital tools. The assessment focused on three functional digital competencies and their levels: C1L1 (uses basic functions of digital devices and understands its importance), C1L2 (uses applications on digital devices), and C2L1 (applies basics of digital data collection and understands its importance).

Each of these three competency areas was assessed through 10 MCQs, making a total of 30 questions. Some items had a single correct response, while others had multiple correct options. Each correct response was awarded 1 mark. For multiple-response items, the answer was marked as correct only if all the correct options were selected. No partial marks were awarded. Thus, the maximum attainable score was 30. This all-or-nothing scoring approach for multi-response items ensured objective evaluation but may underestimate partial knowledge.

The ASHA assessment was designed as a baseline-only diagnostic to inform subsequent training design; accordingly, endline assessment and skill demonstrations were not conducted for this cohort. A future implementation phase will include pre-post training comparisons for ASHAs.

Descriptive statistics were computed for each competency level and the composite total score, including means, SDs, medians, and IQRs. Score distributions were assessed for normality using the Shapiro–Wilk test. Performance was characterized using threshold-based analyses at progressively higher cut-offs (≥50%, ≥60%, ≥70%, ≥80%, ≥90%). To assess whether performance differed across competency levels, the Kruskal-Wallis H test was applied, followed by pairwise Mann–Whitney U tests. Inter-competency associations were examined using Spearman’s rank correlation coefficients. Item-level analysis was conducted for each MCQ, reporting the proportion of correct responses to identify specific knowledge gaps. All analyses used Python (SciPy v1.11). Statistical significance was set at α = 0.05.

## Results

### Design

This section describes the results of each step followed in the design phase.

#### Step 1: Literature review

Literature: 56 articles were identified through the literature review. Findings emphasized that digital health literacy, competency frameworks, and digital tools are increasingly recognized as essential for preparing the health workforce to address evolving public health needs. Case studies and systematic reviews show that digital-enabled training can enhance knowledge, skills, and service delivery while helping bridge healthcare gaps. A range of capacity-building approaches was documented, including training programs, workshops, and online courses. At the same time, challenges remain. Key barriers include digital divides, the limited integration of competencies into medical education, and the lack of standardized frameworks and training materials—particularly in low- and middle-income contexts. These gaps highlight the need for sustainable, context-sensitive approaches to workforce capacity-building across health systems.

Frameworks: 11 frameworks, both national and international, were identified based on the selection criteria described in the methodology section. From these 11 frameworks, we identified 4 frameworks that were well-suited for the purposes of our study: (i) Australian Nursing and Midwifery Framework (ANMF), (ii) NHS Digital Competence Framework, (iii) Digital Health Capability framework for AHPs by Victoria Department of Health, and (iv) FRAC.

Supplementary Table S4 (within Supplementary Material S5) presents a systematic comparison of the DHCF against these four frameworks across key dimensions, highlighting how the DHCF distinguishes itself through its explicit focus on FLHWs in low- and middle-income countries (LMIC) settings, task-based competency anchoring via the FRAC method, integrated assessment and content development, and design for institutional embedding within India’s existing government training infrastructure.

#### Step 2: Defining Digital Health Capacity Building

Based on an integration of these three definitions, DHCB was ultimately defined as the process of strengthening the knowledge, skill sets, attitudes, and resources required by healthcare professionals to use digital systems effectively in fulfilling their mandate to provide quality health services.

#### Step 3: Identification of a standardized framework

After a thorough review of the four frameworks, the competency-driven model employed in the FRAC developed by Mission Karmayogi (Table 1) was selected as the preferred method to develop the DHCF. While the FRAC is being implemented by the Government of India at scale across diverse positions and roles, its national applicability in government administration makes it particularly valuable for institutionalizing the DHCF. For FLHWs specifically, the FRAC holds distinct advantages. Their roles are dynamic, requiring diverse tasks and varied skills, which makes measuring performance and digital competencies challenging. The FRAC method addresses this gap by systematically mapping the roles and activities of FLHWs to the competencies required for effective performance, while also allowing competencies to remain consistent but be flexibly mapped across different cadres depending on their role requirements. In addition, the framework supports level-based differentiation, ensuring that competencies are appropriately benchmarked for varying levels of responsibility. By establishing clear standards for knowledge and skills, the FRAC ensures alignment between intended learning outcomes and the actual capabilities required in practice. Another key advantage of the FRAC is its use of a shared language to define tasks, traits, behaviors, knowledge, and reference standards. This shared language enables vertical integration, aligning government strategies with individual competencies, as well as horizontal integration, aligning human resource processes from recruitment to reward. In a jargon-heavy field, such standardization benefits both FLHWs and stakeholders by reducing misunderstandings, improving collaboration, and ensuring consistent interpretation of tasks, guidelines, and outcomes. For FLHWs, who often work with multiple devices and tools, standardized language and competency mapping ease the burden of navigating different systems while facilitating coordination with diverse stakeholders. For these reasons, the FRAC method was identified as the most suitable approach for this study.

#### Step 4: Defining Digital Health Landscape

The three Digital Health ecosystems were leveraged to define our Digital Health Universe: (i) NHS Digital UK, (ii) ABDM, (iii) Uttar Pradesh’s digital health roadmap. These components were contextualized within the broader, evolving landscape of digital health.

#### Step 5: Identifying competencies and developing a digital health competency dictionary

Ten competencies were identified. Building on these 10 identified competencies, a cadre-agnostic and dynamic Digital Health Competency (DHC) Dictionary was developed. The dictionary serves as a comprehensive toolkit, detailing competencies across three categories—functional, behavioral, and domain—each articulated with explicit progression levels. The full core competency dictionary (10 competencies with all proficiency levels and sub-components) is provided in Supplementary Table S1 (Table S1.1 and Table S1.2).

The DHC Dictionary’s flexible design also allowed for expansion beyond its initial 10 competencies to encompass 17 functional, 10 behavioral, and 18 domain competencies, reflecting the evolving scope of digital health practice. It is important to note that the pilot study evaluated only the initial core set of 10 competencies (specifically, the functional competencies C1 and C2 at selected levels). The expanded set (17 functional, 10 behavioral, 18 domain competencies) was developed subsequently and has not yet been evaluated.

#### Step 6: Mapping digital health competencies and then competency-to-workflow mapping

Based on the FRAC-ing process described in the methodology section, digital competencies were mapped to each of the three FLHW cadres according to their specific roles and responsibilities (Supplementary Material 4, Tables S3.1–S3.4). Using the initial version of the DHC (Digital Health Competency) dictionary, competencies were assigned as follows:

- ANMs: 4 digital competencies spanning 6 competency levels (C1L1, C1L2, C2L1, C2L2, C3L1, C5L1)
- ASHAs: 4 digital competencies spanning 4 competency levels (C1L1, C1L2, C2L1, C5L1)
- AWWs: 2 digital competencies ranging across 2 competency levels (C1L1, C1L2)

**Figure 4:**
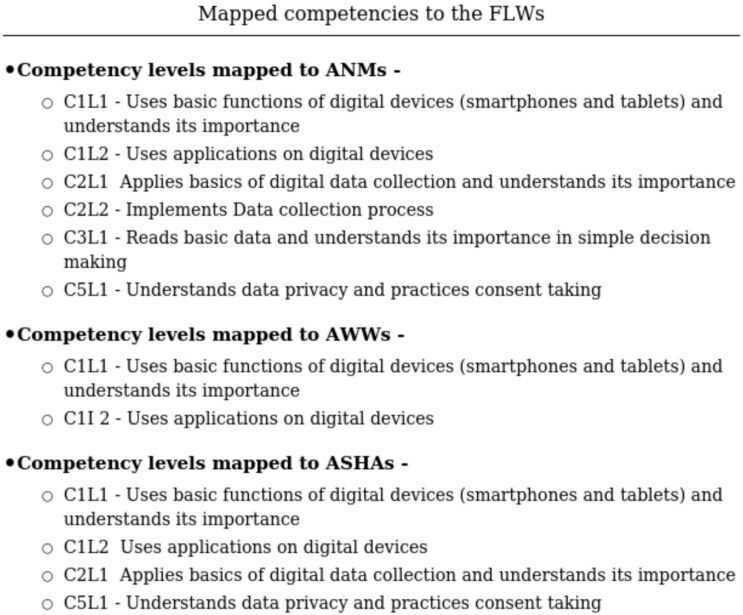
Mapped competencies to the FLHWs.

The specific mapping of competencies with the three FLHWs is shown below in Fig. 4**.** This role-specific mapping enabled the design of tailored training curricula based on digital skills most relevant to each cadre’s functional responsibilities. The competency framework distinguishes between competency areas (C1, C2, C3, C5) and competency levels (L1, L2) to ensure appropriate skill progression. This initial mapping was based on the first version of the DHC dictionary. Over time, FLHWs have been comprehensively re-mapped using an updated and refined dictionary that reflects evolving digital health requirements and more nuanced role definitions.

Table 2 maps the competency domains to typical FLHW digital workflows, the digital tools involved, common failure points encountered in practice, and how the corresponding competencies mitigate these barriers. This mapping illustrates the practical anchoring of the DHCF in real-world frontline digital health tasks.

**Table 2:** Mapping of competency domains to FLHW digital workflows and common failure points.

Competency domain	Typical FLHW tasks	Digital tools	Common failure points	How competency mitigates
C1: Using digital devices and applications	Device startup, app login, navigation, ABHA registration	e-Kavach, ABHA app, RCH portal	Authentication failures, forgotten passwords, inability to navigate menus	Builds foundational device handling and app navigation skills
C2: Data collection and management	Patient registration, immunization records, ANC data entry	e-Kavach, UP-HMIS, NCD-GOI ANM	Connectivity-dependent sync failures, duplicate paper-digital entry, data loss	Teaches data entry workflows, offline/online sync concepts, error checking
C3: Data analysis and communication	Estimating drug/vaccine requirements, reviewing dashboards	HMIS dashboards, Excel reports	Inability to interpret charts, over-reliance on paper registers	Builds ability to read and interpret basic data for decision-making
C5: Data privacy and consent	OTP verification for ABHA, patient consent taking	ABHA app, e-Kavach consent modules	Pressure to meet registration targets overrides consent practices, unclear OTP process	Establishes understanding of digital consent, privacy principles, and ethical data handling

#### Step 7: Development of the Digital Health Assessment Framework

The Digital Health Assessment Framework comprises 250 MCQs covering seven core digital competencies. The MCQ-based assessment by competency and level, together with representative sample items, is provided in Supplementary Material S3 (Supplementary Table S2). The skill-based components of competencies were tested using an observation checklist (provided in full in Supplementary Material S3).

#### Step 8: Review and feedback

##### Domain experts

The review and feedback of the framework from the domain experts highlighted specific refinements to the competency list (C1–C7) and their mapping to ANMs’ roles. For example, under the competency C1—Using digital devices and applications, reviewers noted an overlap in Level 2 items, suggesting a clearer separation between installation/uninstallation of applications and the use of common apps, with login/password creation to be integrated accordingly. Reviewers also highlighted that data collection and management using digital devices—competency C2- was critical for ANMs, with at least Levels 1–2 recommended for them, given ANMs’ central role in recording and reporting community health data.

Similarly, C3—Data analysis and communication, Levels 1–2, were identified as necessary to enable ANMs to interpret population-level data, particularly in programs like POSHAN Abhiyaan. C7—Digital health information systems and apps also emerged as highly relevant, with Levels 1–3 recommended, since ANMs are directly responsible for services such as immunization, antenatal care, and family planning, all of which rely on HIS platforms. Additionally, feedback supported inclusion of C1L3—interoperable system use, C4L1–L2—digital communication for teaching, learning, and self-development, and C5L1–2—basic data management practices. Tools such as e-Kavach, already implemented through ASHAs, further reinforced the applicability of C7 to frontline workers. Overall, this feedback informed a more precise alignment of competencies with ANMs’ functional responsibilities across service delivery, data collection, and digital communication.

It is important to note that these findings are based on an earlier version of the DHC dictionary; subsequent updates may have refined the scope and labeling of C1–C7, although the underlying principles of alignment with ANM roles remain relevant.

##### Auxiliary nurse midwives

Feedback from ANMs of PHC Kursi in Barabanki and CHC Sohawal and CHC Karnailganj in Ayodhya and Gonda, respectively, revealed the following insights that were later utilized in optimizing the DHC dictionary.

FLHWs displayed varying levels of digital proficiency across competencies. For C1—*Using Digital Devices and Applications*, formal training on basic device use is often limited or informal, with app-specific training (e.g. eKavach) providing only superficial coverage. Younger ANMs tend to be more adept, while older ANMs often rely on peers or family for assistance. ASHAs and AWWs, in particular, struggle with tasks such as installing or uninstalling apps on supplied tablets, and knowledge of features like GPS or Bluetooth remains low.

In C2—*Data Collection and Management*, most cadres are comfortable with drop-down menus, calendars, and input formats, though ASHAs and AWWs face greater challenges than ANMs. Ensuring accuracy at the point of collection is critical, as errors cannot be corrected once data is synced, and feedback highlights the need for efficiency improvements and workflow harmonization to reduce duplication. In *Data Analysis and Communication*, ANMs draw on data for decision-making, such as estimating drug and vaccine requirements, but this practice largely predates digital tools. Digital platforms for analysis are seldom fully trusted or integrated, with paper registers still serving as the primary reference, and examples of meaningful digital impact remain limited. In *Data Privacy and Consent—C5*, ANMs demonstrate a basic understanding but apply it more as a moral practice than through formal guidelines. While clinical consent is routinely sought, digital consent, such as OTP verification for ABHA registration, presents challenges, especially under pressure to meet target numbers. This could be due to the fact that training on privacy is briefly covered during ANM induction, but is not reinforced thereafter.

Overall, digital training remains short and app-specific, with little alignment to the time and effort required for real-world fieldwork, often leading to duplication of work both online and offline. FLHWs emphasized that digital competencies would become meaningful only when digital tools reduce workloads, and they suggested more practical training on tablets, typing, and age-tailored programming.

#### Step 9: Creating competency-mapped content

Content sets were developed as PPT presentations for each Cadre of FLHWs, called *Digital Health Capacity Building packages.* The content sets were converted into audio-visual content, which could be hosted on any digital platform, and encompassed a range of key themes relevant to the roles and responsibilities of all three cadres. These included:

1) Basic functions of digital devices and their importance
2) Applications on digital devices
3) Basics of digital data collection and its importance
4) Data collection process
5) Data privacy and practices consent taking
6) Basic data and its importance in simple decision-making

**Figure 5:**
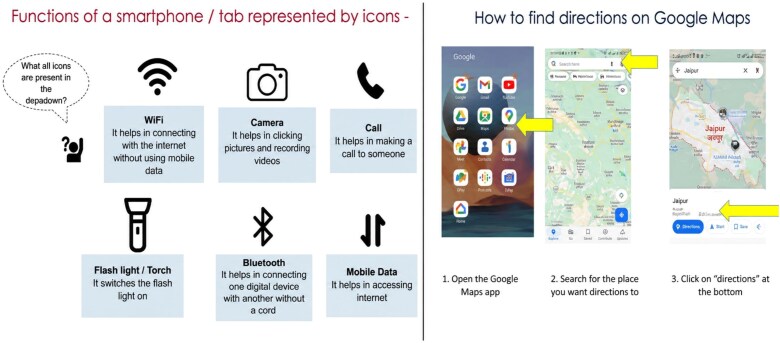
Slides of content on basic functions of digital devices and their importance and applications on digital devices.

**Figure 6:**
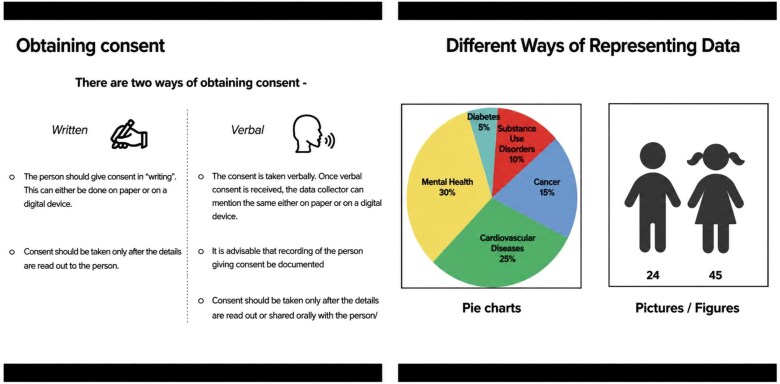
Slides of content on consent taking and ways of representing data.

Examples of the content developed for these modules are displayed in Figs. 5 and 6 below.

### Implementation

The DHCB delivery process was effectively operationalized through a combination of role-based training design, assessments, and integrated delivery mechanisms.

#### Delivery process and mechanism

To put the Delivery Process and Mechanism into practice, the UPTSU piloted the integration of the DHCB modules within broader competency development initiatives. For instance, the existing e-Kshamata LMS was leveraged to facilitate the training, which was delivered through both web-based and Android application-based interfaces.

#### Institutionalization

The Government of UP and UPTSU have been integrating the ASHA digital literacy modules into ASHA induction training and cluster meetings, resulting in state-wide coverage and reaching 160 000 ASHA workers. This is the project’s first step towards institutionalizing the DHCF.

### Evaluation

#### Monitoring learner engagement and feedback

The DHCB framework places equal emphasis on governance oversight and real-time monitoring to ensure training efforts translate into measurable outcomes. A digital dashboard embedded within the LMS will serve as the central monitoring tool, providing live insights on staff onboarding, user engagement module completion, assessment scores, and certification rates. With state, district, and facility-level access, these dashboards create visibility across the system and enable timely course corrections. In parallel, operational data from existing health systems (e.g. quality of digital records, frequency of data use) will be tracked to assess whether competencies are being applied in day-to-day practice. The DHCB competency dictionary and related knowledge products & assessments will go through periodic revisions and updates based on the user engagement data & feedback.

User engagement will be leveraged not just as a metric but as a driver of program success. Structured mechanisms—monthly district reviews led by CMOs, quarterly state-level reviews chaired by the Steering Committee, and cadre-specific working group inputs—anchor accountability while also fostering recognition for high-performing regions. To complement this, multi-channel feedback loops such as WhatsApp peer groups (during training), Interactive Voice Response System (IVRS) feedback (post-certification), and support by Tech Mitras, a nodal person to support and resolve any doubts at the block level, provide continuous, bottom-up signals on training relevance, usability, and challenges.

The monitoring process is learning-driven rather than compliance-driven. By blending real-time data with human feedback, the program remains adaptive, user-centric, and focused on outcomes—ensuring that DHCB directly strengthens service delivery across the health system.

#### Pilot study with ANMs

##### Participant overview

Seventy ANMs were enrolled across two districts (Barabanki: *n* = 32; Sitapur: *n* = 38). Participation in each assessment component varied due to scheduling constraints and the phased delivery of training modules across weekly meetings. The number of ANMs with paired baseline-endline data ranged from 40 (C2L1) to 58 (C1L1), depending on the competency level.

##### Knowledge assessment results

Table 3 presents the descriptive statistics and inferential test results for paired baseline-endline knowledge assessments across all four competency levels. Results are reported for the paired analytical sample (ANMs with both baseline and endline scores for each respective competency level).

**Table 3:** Paired baseline-endline knowledge assessment results by competency level (Wilcoxon signed-rank test).

Competency level	*n*	Baseline mean (SD)	Endline mean (SD)	Baseline median (IQR)	Endline median (IQR)	Mean Δ (SD)	Median Δ	W	*P*-value	Effect size *r* (d)
**C1L1: Device basics**	58	7.78 (1.91)	8.36 (1.96)	8.0 (6.2–9.0)	9.0 (8.0–9.8)	+0.59 (1.81)	+1.0	152.5	<.001^***^	0.61 (0.32)
**C1L2: Applications**	57	8.02 (2.83)	8.11 (2.79)	9.0 (7.0–10.0)	9.0 (8.0–10.0)	+0.09 (0.95)	0.0	174.5	.490	0.14 (0.09)
**C2L1: Data collection basics**	40	5.78 (1.44)	7.22 (1.62)	6.0 (5.0–7.0)	8.0 (6.0–8.0)	+1.45 (1.48)	+1.5	57.0	<.001^***^	0.84 (0.98)
**C2L2: Data collection process**	41	7.66 (1.80)	7.98 (2.20)	8.0 (6.0–9.0)	9.0 (7.0–10.0)	+0.32 (2.18)	+1.0	192.5	.109	0.31 (0.15)

W = Wilcoxon signed-rank test statistic; r = rank-biserial correlation; d = Cohen’s d. ^***^P < .001. All scores out of a maximum of 10.

**C1L1: Using basic functions of digital devices**

Among 58 ANMs with paired data, the mean knowledge score increased from 7.78 (SD = 1.91) at baseline to 8.36 (SD = 1.96) at endline, a statistically significant improvement (Wilcoxon W = 152.5, *P* < .001). The median score improved from 8.0 (IQR: 6.2–9.0) to 9.0 (IQR: 8.0–9.8). Of the 58 ANMs, 31 (53.4%) showed improvement, 8 (13.8%) showed decline, and 19 (32.8%) maintained the same score. The rank-biserial effect size was *r* = 0.61, indicating a large effect. The proportion scoring ≥80% increased from 67.2% to 86.2%, and the proportion scoring ≥90% increased from 48.3% to 60.3%.

***C1L2: Using applications on digital devices***

Among 57 ANMs with paired data, baseline scores were already high (mean = 8.02, SD = 2.83; median = 9.0), indicating a ceiling effect. The mean endline score was 8.11 (SD = 2.79), representing a non-significant change (Δ = +0.09, Wilcoxon W = 174.5, *P* = .490). An equal number of ANMs showed improvement and decline (14 each), with 29 (50.9%) unchanged. The rank-biserial effect size was small (*r* = 0.14). The proportion scoring ≥90% was 68.4% at baseline and 73.7% at endline.

***C2L1: Basics of digital data collection***

This competency showed the largest and most consistent improvement. Among 40 ANMs with paired data, the mean score increased from 5.78 (SD = 1.44) to 7.22 (SD = 1.62), representing a mean change of +1.45 (SD = 1.48). This improvement was highly significant (Wilcoxon W = 57.0, *P* < .001), with a large effect size (*r* = 0.84, Cohen’s d = 0.98). Thirty-two (80.0%) of ANMs showed improvement, 5 (12.5%) showed decline, and 3 (7.5%) were unchanged. The proportion scoring ≥80% increased substantially from 10.0% at baseline to 60.0% at endline, and the proportion scoring ≥90% increased from 2.5% to 17.5%. This competency had the lowest baseline scores and the greatest room for improvement.

***C2L2: Implementing the data collection process***

Among 41 ANMs with paired data, the mean score increased from 7.66 (SD = 1.80) to 7.98 (SD = 2.20), a non-significant change (Wilcoxon W = 192.5, *P* = .109). However, 23 (56.1%) of ANMs showed improvement, 10 (24.4%) showed decline, and 8 (19.5%) were unchanged. The rank-biserial effect size was small-to-medium (*r* = 0.31). The proportion scoring ≥90% increased from 39.0% to 51.2%.

##### Threshold-based analysis

Table 4 presents the proportion of ANMs meeting performance thresholds at baseline and endline.

**Table 4:** Proportion of ANMs meeting performance thresholds at baseline and endline.

Competency level	*n* (paired)	Baseline ≥80%	Endline ≥80%	Baseline ≥90%	Endline ≥90%	Δ in ≥90% (%pts)
C1L1	58	67.2%	86.2%	48.3%	60.3%	+12.1
C1L2	57	73.7%	80.7%	68.4%	73.7%	+5.3
C2L1	40	10.0%	60.0%	2.5%	17.5%	+15.0
C2L2	41	68.3%	68.3%	39.0%	51.2%	+12.2

Scores out of 10. ≥80% = ≥8/10; ≥90% = ≥9/10.

##### District-level analysis

Table 5 presents district-stratified results for knowledge assessments.

**Table 5:** District-stratified knowledge assessment results.

Competency	District	*n*	Baseline mean (SD)	Endline mean (SD)	Mean Δ	W	*P*-value
**C1L1**	Barabanki	26	7.96 (1.71)	8.38 (1.88)	+0.42	39.0	.073
	Sitapur	32	7.62 (2.07)	8.34 (2.05)	+0.72	34.5	.002^**^
**C1L2**	Barabanki	29	7.41 (3.37)	7.62 (3.39)	+0.21	27.5	.184
	Sitapur	28	8.64 (2.06)	8.61 (1.95)	−0.04	54.5	.740
**C2L1**	Barabanki	16	5.50 (1.32)	7.62 (1.41)	+2.12	0.0	<.001^***^
	Sitapur	24	5.96 (1.52)	6.96 (1.71)	+1.00	42.0	.005^**^
**C2L2**	Barabanki	18	7.94 (1.70)	8.28 (1.87)	+0.33	42.0	.293
	Sitapur	23	7.43 (1.88)	7.74 (2.45)	+0.30	59.5	.251

^**^ *P* < .01; ^***^P < .001.

District-level analysis revealed that improvements were consistent in direction across both districts, though they reached statistical significance in only some district-competency combinations. C2L1 showed significant improvements in both Barabanki (W = 0.0, *P* < .001) and Sitapur (W = 42.0, *P* = .005). C1L1 reached significance in Sitapur (*P* = .002) but not Barabanki (*P* = .073), likely reflecting lower statistical power in the smaller Barabanki subsample. C1L2 and C2L2 showed non-significant changes in both districts, consistent with the pooled analysis.

##### Skill demonstration results

Skill demonstrations were completed by 36 ANMs for C1 (L1 + L2) and 35 ANMs for C2L1. Skill demonstrations were conducted after both weeks of training were completed; not all ANMs who completed knowledge assessments were available for skill demonstrations due to scheduling conflicts (Table 6).

**Table 6:** Skill demonstration assessment results.

Competency	Max score	District	*n*	Mean (SD)	Median (IQR)	% ≥80%	% ≥90%	≥90% count
**C1 (L1 + L2)**	9	Barabanki	18	8.39 (0.70)	9.0 (8.0–9.0)	94.4%	55.6%	10/18
		Sitapur	18	7.94 (2.26)	9.0 (8.0–9.0)	77.8%	61.1%	11/18
		Combined	36	8.17 (1.65)	9.0 (8.0–9.0)	86.1%	58.3%	21/36
**C2L1**	14	Barabanki	17	11.76 (1.09)	12.0 (11.0–13.0)	100%	23.5%	4/17
		Sitapur	18	11.17 (3.17)	12.0 (10.8–13.0)	88.9%	33.3%	6/18
		Combined	35	11.46 (2.35)	12.0 (11.0–13.0)	94.3%	28.6%	10/35

≥80% defined as ≥7.2/9 for C1 and ≥11.2/14 for C2L1. ≥90% defined as ≥8.1/9 for C1 and ≥12.6/14 for C2L1.

For C1 (L1 + L2), the combined mean skill demonstration score was 8.17 out of 9 (90.7%), with 21 of 36 ANMs (58.3%) scoring ≥90% and 31 of 36 (86.1%) scoring ≥80%. In Barabanki, 10 of 18 ANMs (55.6%) scored ≥90%; in Sitapur, 11 of 18 (61.1%) achieved this threshold.

For C2L1, the combined mean score was 11.46 out of 14 (81.8%), with 10 of 35 ANMs (28.6%) scoring ≥90% and 33 of 35 (94.3%) scoring ≥80%. These results suggest strong practical skill transfer for basic device operations (C1) and solid foundational skill in data collection workflows (C2L1), with data collection showing room for further skill development.

##### Summary of findings

Table 7 provides an integrated summary across all assessment modalities.

**Table 7:** Integrated summary of knowledge and skill assessment results.

Competency level	Knowledge baseline (mean/10)	Knowledge endline (mean/10)	Significance	Effect size	Skill demo (mean/%)	Interpretation
C1L1: Device basics	7.78	8.36	*P* < .001	Large (*r* = 0.61)	8.17/9 (90.7%)	Significant gain; high skill transfer
C1L2: Applications	8.02	8.11	*P* = .490	Small (*r* = 0.14)	— (combined with C1L1)	Ceiling effect; already high baseline
C2L1: Data collection	5.78	7.22	*P* < .001	Large (*r* = 0.84)	11.46/14 (81.8%)	Largest gain; strong improvement
C2L2: Data process	7.66	7.98	*P* = .109	Small (*r* = 0.31)	— (not assessed)	Modest improvement; not significant

Effect size interpretation: small (r < 0.3), medium (r = 0.3–0.5), large (r > 0.5).

The pilot assessment of ANMs yielded several important findings. First, the training intervention produced statistically significant and practically meaningful improvements in two of the four competency levels assessed: C1L1 (basic device use; *P* < .001, *r* = 0.61) and C2L1 (data collection basics; *P* < .001, *r* = 0.84). The C2L1 improvement was the largest observed, with a Cohen’s d of 0.98, representing a near-doubling of the proportion of ANMs meeting the 80% performance threshold (from 10.0% to 60.0%).

Second, two competency levels (C1L2 and C2L2) did not show statistically significant improvements. For C1L2, this is attributable to a ceiling effect: baseline scores were already high (median = 9.0/10), leaving limited room for measurable improvement. For C2L2, the non-significant result may reflect both the higher complexity of data collection process tasks and the limited training time available within the 1-h weekly meeting format.

Third, skill demonstration results were strong, with 86.1% of assessed ANMs scoring ≥80% on C1 device operations and 94.3% scoring ≥80% on C2L1 data collection tasks. This suggests effective transfer of knowledge into practical skills, particularly for fundamental device operations.

Fourth, district-level analysis showed broadly consistent patterns across Barabanki and Sitapur, though the magnitude of improvement varied. C2L1 showed statistically significant improvements in both districts, while C1L1 reached significance only in Sitapur. These differences likely reflect both sample size differences and baseline variability between sites rather than systematic site-level differences in training effectiveness.

Finally, the variable completion rates across competency levels (ranging from *n* = 40 to *n* = 58 for paired assessments, against 70 enrolled) highlight the practical challenges of conducting assessments within routine health system meetings. This underscores the need for flexible, blended assessment approaches in future implementations.

Based on the pilot, we identified several lessons for implementation. First, institutional support emerged as a key enabler for effective training delivery. Competing clinical responsibilities frequently led to the deprioritization of DHCB sessions. Second, the pilot highlighted challenges related to the scalability and sustainability of the in-person training model. Third, findings on assessment design revealed that ANMs were more comfortable with practical, demonstration-based assessments. Finally, the pilot uncovered encouraging examples of peer learning, with younger ANMs supporting senior colleagues. These insights, feedback, and observations obtained from the ANM pilot sessions enabled iterative enhancement of the FLHWs’ training package for further implementation. Additionally, the pilot promoted the development of long-term stakeholder relationships with local authorities, supporting ongoing and future implementation of the digital health capacity-building initiative. Finally, the pilot findings also informed the recommendation that the DHCF be implemented using a minimum viable blended training approach (Supplementary Appendix C).

#### Baseline assessment with accredited social health activists

All 32 ASHAs completed the assessment in full, with no missing data across any competency level (*n* = 32 for all analyzes). Table 8 presents the descriptive statistics for baseline knowledge scores across all three competency levels and the composite total.

**Table 8:** ASHA baseline knowledge assessment: descriptive statistics by competency level.

Competency	*n*	Mean (SD)	Median (IQR)	Range	Mean %	Score = 0	≥50%	≥60%	≥70%
**C1L1: Device basics**	32	4.66 (2.56)	5.0 (3.0–6.2)	0–9	46.6%	3 (9.4%)	17 (53.1%)	14 (43.8%)	8 (25.0%)
**C1L2: Applications**	32	4.06 (2.91)	4.0 (2.0–6.2)	0–9	40.6%	6 (18.8%)	13 (40.6%)	9 (28.1%)	8 (25.0%)
**C2L1: Data collection**	32	3.25 (2.08)	3.5 (1.0–5.0)	0–6	32.5%	4 (12.5%)	12 (37.5%)	5 (15.6%)	0 (0.0%)
**Total (/30)**	32	11.97 (6.64)	12.5 (9.0–16.0)	0–24	39.9%	3 (9.4%)	11^a^ (34.4%)	4^b^ (12.5%)	—

All competency-level scores out of 10. Total out of 30.

^a^Total ≥50% = ≥15/30.

^b^Total ≥60% = ≥18/30.

Overall performance was low across all three competency levels. The mean total score was 11.97 out of 30 (39.9%), with a median of 12.5 (IQR: 9.0–16.0). No ASHA achieved a total score above 24/30 (80%), and only 4 ASHAs (12.5%) scored ≥60% overall. Three ASHAs (9.4%) scored 0 across all three competencies, suggesting inability to engage meaningfully with the assessment.

Performance declined across the competency hierarchy. C1L1 (basic device use) was the strongest competency, with a mean of 4.66/10 (46.6%); 53.1% of ASHAs scored ≥50%, and 25.0% scored ≥70%. C1L2 (using applications) showed a wider spread (SD = 2.91) with a higher proportion of zero scores (18.8%) but also a small cluster of high performers (9.4% scoring ≥90%). C2L1 (data collection) was the weakest competency: the mean was 3.25/10 (32.5%), no ASHA scored above 6/10, and only 15.6% reached the 60% threshold (Table 9).

**Table 9:** Frequency distribution of ASHA baseline scores by competency.

Score range (/10)	C1L1 *n* (%)	C1L2 *n* (%)	C2L1 *n* (%)
**0**	3 (9.4%)	6 (18.8%)	4 (12.5%)
**1–2**	4 (12.5%)	4 (12.5%)	8 (25.0%)
**3–4**	8 (25.0%)	9 (28.1%)	8 (25.0%)
**5–6**	9 (28.1%)	5 (15.6%)	12 (37.5%)
**7–8**	6 (18.8%)	5 (15.6%)	0 (0.0%)
**9–10**	2 (6.2%)	3 (9.4%)	0 (0.0%)

C2L1 scores were truncated at 6/10, with no ASHA scoring above this level.

A Kruskal–Wallis test across the three competency levels was non-significant (H = 4.49, *P* = .106). However, pairwise Mann–Whitney U tests revealed that C2L1 scores were significantly lower than C1L1 scores (U = 679.5, *P* = .024), while the C1L1 vs C1L2 (U = 578.5, *P* = .372) and C1L2 vs C2L1 (U = 579.5, *P* = .364) comparisons were non-significant. This confirms that digital data collection knowledge was significantly weaker than basic device knowledge.

Spearman’s rank correlations between competency scores were all positive and statistically significant: C1L1–C1L2 (ρ = 0.694, *P* < .001), C1L1–C2L1 (ρ = 0.558, *P* < .001), and C1L2–C2L1 (ρ = 0.595, *P* < .001). These moderate-to-strong correlations indicate that digital health knowledge partly reflects a general underlying capacity, though the weaker and truncated C2L1 distribution suggests that data collection also draws on distinct knowledge domains not captured by device familiarity alone.

Item-level analysis identified specific knowledge gaps within each competency. For C1L1, the strongest items were Q7 (83.9% correct) and Q8 (74.2%), while Q10 (16.1%) and Q3 (25.8%) were weakest. For C1L2, performance was uniformly low, with 6 of 10 items below 50% correct and Q1, Q7, and Q8 each at 25.8%. For C2L1, Q3 was answered correctly by 67.7%, but Q4 and Q9 had only 6.5% correct response rates, indicating near-complete absence of knowledge in those areas (likely related to data integrity and synchronization concepts). Full item-level results are presented in Table 10.

**Table 10:** Item-level analysis: proportion of correct responses by question.

Question	C1L1% correct	C1L2% correct	C2L1% correct
**Q1**	57.6%	25.8%	29.0%
**Q2**	54.5%	**61.3%**	22.6%
**Q3**	25.8%	**71.0%**	**67.7%**
**Q4**	41.9%	41.9%	6.5%
**Q5**	**61.3%**	45.2%	38.7%
**Q6**	35.5%	51.6%	12.9%
**Q7**	**83.9%**	25.8%	51.6%
**Q8**	**74.2%**	25.8%	51.6%
**Q9**	32.3%	41.9%	6.5%
**Q10**	16.1%	29.0%	48.4%

Values in bold exceed 60%. Values below 30% are flagged as critical gaps. Based on ~n = 31 respondents per item (slight variation due to unanswered items

## Discussion

This feasibility study sought to design, develop, and evaluate a theory-driven, evidence-informed, and scalable DHCF for FLHWs in Uttar Pradesh. Using a rigorous, multi-stage process, the FRAC methodology was adapted to systematically identify and map digital health competencies across key FLHW cadres, including ANMs, ASHAs, and AWWs. This approach resulted in a cadre-agnostic digital health competency dictionary, complemented by competency-aligned training content and assessment tools to support structured capacity building. Feasibility evaluation through an ANM pilot and ASHA baseline assessment demonstrated both the relevance of the framework and the persistent gaps in foundational digital competencies, while also highlighting the importance of institutional support and scalable, hybrid delivery models for workforce training.

This study extends the existing literature on DHCFs in several important ways. First, it applies the FRAC methodology to digital health, offering a systematic mechanism to link competencies directly to roles and routine activities. Unlike prior frameworks that rely primarily on expert-driven mapping exercises to identify potential competencies, the FRAC approach anchors competencies in day-to-day tasks, thereby enhancing their practical relevance and applicability. This task-based linkage addresses a key limitation of earlier frameworks, including DECODE, where competencies are often articulated at a high level and may be difficult to operationalize in frontline contexts (Car et al. 2025). Second, while most existing frameworks focus on clinicians working in hospital or facility-based settings, this study foregrounds the unique digital competency requirements of FLHWs operating in rural and community-based environments in LMICs. Among existing frameworks, only a limited number, such as TIGER, include empirical evidence from LMICs, underscoring the contribution of this study in addressing a critical evidence gap (Nazeha et al. 2020). Third, the DHCF moves beyond binary or single-level competency definitions by incorporating a multi-tiered progression model. This enables more nuanced assessment of workforce readiness and supports personalized learning pathways.

### Tool usability as a complementary dimension

While the DHCF addresses the user capability side of the digital health equation, our findings—particularly the ANM feedback on workflow duplication, data sync limitations, and inability to correct synced data—strongly suggest that poor tool design constitutes a major barrier that training alone cannot overcome. ANMs reported specific User Interface/User experience (UI/UX) challenges including non-intuitive menu navigation, unclear error messages during data sync failures, and the persistence of dual paper-digital workflows that increase rather than reduce workload. These tool-side barriers interact with and compound competency gaps: even a well-trained FLHW cannot perform effectively with a poorly designed tool, and a well-designed tool remains underutilized without adequate competencies.

We therefore propose a phased approach to digital health optimization: Phase 1 (current)—competency building using the DHCF; Phase 2—integration of UI/UX assessment and participatory tool redesign informed by competency data and FLHW feedback; Phase 3—measurement of the combined impact of improved tools and improved competencies on retention and service delivery outcomes. This framing positions the DHCF as one component of a broader digital health optimization system that addresses both user capability and tool quality.

### Equity, pre-digital scaffolding, and safeguards against punitive use

The ASHA baseline results reveal substantial foundational digital skill gaps. The mean total score was 11.97/30 (39.9%), with a median of 12.5 (IQR: 9.0–16.0). Only 12.5% of ASHAs scored ≥60% overall, and none achieved above 80%. Performance declined systematically across the competency hierarchy: C1L1 (device basics, 46.6%) > C1L2 (applications, 40.6%) > C2L1 (data collection, 32.5%), with C2L1 scores significantly lower than C1L1 (Mann–Whitney U = 679.5, *P* = .024). The C2L1 distribution was truncated at 6/10, and item-level analysis revealed near-zero knowledge on items related to data integrity and synchronization (Q4 and Q9: 6.5% correct). Three ASHAs (9.4%) scored 0 across all competencies, and the proportion scoring 0 on individual competencies ranged from 9.4% (C1L1) to 18.8% (C1L2). These findings suggest that many ASHAs require pre-digital scaffolding—including basic literacy and numeracy support, device navigation practice (turning on/off, unlocking, identifying icons), and authentication concepts (passwords, OTPs)—before app-specific digital health training can be effective. We recommend that the DHCF implementation pathway incorporate a readiness assessment at entry, with participants below a defined threshold routed to a pre-digital scaffolding module before entering the competency-based training pathway. Supportive supervision and mentoring, rather than standalone training events, are essential complements to this approach.

It is also critical to address the risk that competency frameworks could be used punitively—for example, linking assessment scores to performance management, incentive allocation, or disciplinary action without ensuring adequate system enablers. Capacity-building programs can only be meaningfully customized to individual competency gaps if FLHWs engage with baseline assessments constructively, without fear of adverse consequences. We therefore argue that competency assessment should be formative rather than summative in the initial implementation phase. Organizations and decision-makers must recognize competency-based capacity building not as a one-time training event but as a continuous developmental process—spanning competency mapping, baseline assessment, targeted training, and progression toward competency certification.

Any linkage to performance management must be conditional on the provision of adequate system enablers: functional devices of appropriate quality, reliable network connectivity, manageable workloads that account for digital tasks, training delivered in the worker’s primary language, and sufficient time allocation for digital documentation. Without these enablers, poor competency scores may reflect system failures rather than individual deficits, and punitive application could exacerbate inequities rather than address them. Conversely, positive incentive mechanisms—such as recording competency certifications in official service records and linking them to professional development pathways—can serve as constructive nudges that make training programs appealing to healthcare staff, fostering voluntary engagement rather than compliance-driven participation.

### ASHA baseline findings and implications for training design

The ASHA baseline assessment provides the first empirical characterization of digital health knowledge among this cadre in Uttar Pradesh and yields several findings with direct implications for the design of competency-based training.

First, the observed competency hierarchy—C1L1 (46.6%) > C1L2 (40.6%) > C2L1 (32.5%)—is pedagogically meaningful. The progression from concrete device familiarity to abstract data collection concepts mirrors established learning hierarchies and suggests that training design should follow this sequence, building from device basics through application use to data collection workflows. The significant difference between C1L1 and C2L1 scores (*P* = .024) confirms that these are empirically distinct competency domains, despite their moderate positive correlation (ρ = 0.558), validating the DHCF’s multi-level structure.

Second, the C2L1 results are particularly concerning given the increasing reliance on ASHAs for digital data collection through platforms such as e-Kavach and ABDM-linked applications. No ASHA scored above 60% on C2L1, and item-level analysis identified near-complete gaps in knowledge related to data integrity and synchronization (6.5% correct on two items). These findings suggest that current deployment of data collection tools substantially outpaces workforce readiness, with direct implications for the quality and reliability of digitally captured health data. Training programs must allocate disproportionate time and emphasis to data collection competencies, ideally using hands-on practice with the actual tools ASHAs use in the field.

Third, the wide performance spread (SD = 2.08–2.91 across competencies; total scores ranging from 0 to 24/30) highlights significant heterogeneity within the ASHA workforce. The bimodal distribution observed in C1L2—with clusters at zero and at moderate scores—suggests the coexistence of two distinct subgroups: a group with no app-related knowledge and another with partial familiarity, likely reflecting differential exposure to smartphones and digital tools in their personal and professional lives. A one-size-fits-all training approach is therefore unlikely to be effective. The DHCF’s tiered competency structure provides a natural mechanism for differentiated learning pathways, where the entry-level readiness assessment can route ASHAs to the appropriate starting module. Furthermore, ANM baseline scores on C1L1 (7.78/10) were substantially higher than ASHA baseline scores on the same competency (4.66/10), underscoring the differential starting points and the need for cadre-specific training pathways.

Fourth, the significant positive inter-competency correlations (ρ = 0.56–0.69, all *P* < .001) indicate that digital health knowledge partly reflects a general underlying capacity—ASHAs who perform better on one competency tend to perform better on others. However, the truncated C2L1 distribution (maximum score 6/10 vs 9/10 for C1L1 and C1L2) confirms that data collection also involves distinct knowledge that requires targeted training beyond general digital device familiarity. This finding supports the DHCF’s separation of device operation (C1) and data management (C2) as distinct competency domains.

These baseline results serve a dual purpose within the DHCF development process: they validate the framework’s diagnostic utility by identifying specific, measurable competency gaps, and they directly inform the content, sequencing, and delivery strategy for ASHA-specific capacity-building programs. The planned next phase will evaluate the DHCF’s effectiveness in improving these competencies through a pre-post training design. The absence of demographic data for this cohort precludes analysis of whether age, education, or years of service mediate digital competency levels, and this should be addressed in future assessments.

The study demonstrates several methodological and implementation strengths. By embedding assessment within the framework development process, the DHCF integrates evaluation as a core design element rather than a downstream activity. Small-scale pilots and baseline assessments were leveraged to identify competency gaps and generate early feedback, allowing iterative refinement of the framework. In addition, the participatory approach adopted during framework development strengthened its face validity and feasibility. Furthermore, the study emphasizes sustainability by proposing integration of the DHCF into existing health system mechanisms, such as cluster meetings, induction training, and national digital learning platforms supported by ABDM and the National Health Authority. This systems-aligned approach reduces duplication and increases the likelihood of institutional adoption.

This study is framed as a feasibility and proof-of-concept evaluation, and findings should be interpreted accordingly. Despite these strengths, several limitations warrant consideration. The absence of a control group precludes causal attribution of improvements to the training intervention; practice effects, test familiarity, and secular trends cannot be ruled out. Baseline and endline MCQ sets were matched but not identical, which may introduce differential difficulty effects. Variable completion rates across competency levels resulted in different analytical samples for each comparison, potentially introducing selection bias. The study was conducted in only two districts and does not fully capture the heterogeneity of digital maturity, infrastructure, and socio-economic contexts across India or other LMICs, thus limiting generalizability. External validation in diverse settings is therefore necessary. For the ASHA baseline assessment, no demographic data (age, education, years of service) were collected, precluding analysis of associations between personal characteristics and digital competency levels. The stringent all-or-nothing scoring rubric for multi-response MCQ items may underestimate partial knowledge, particularly for C2L1 where several items required multiple correct selections. Additionally, the ASHA assessment was conducted at a single facility (CHC Satrikh, Barabanki), and the sample size (*n* = 32) limits both generalizability and the statistical power of comparative analyzes. Furthermore, while the framework focuses on measurable digital competencies, it does not fully capture the complex socio-emotional and relational skills required by FLHWs. Future research should explore mixed-methods and performance-based assessment approaches. Inter-rater reliability for skill demonstrations was not formally assessed in this pilot, which limits the confidence in skill assessment results; future studies should incorporate formal inter-rater reliability testing. Finally, the study does not assess long-term competency retention, an important consideration given FLHWs’ high workloads and competing priorities. These limitations are consistent with the study’s framing as a feasibility and proof-of-concept evaluation rather than a definitive effectiveness trial.

### Future evaluation roadmap

Building on the feasibility evidence from this study, we propose a four-phase evaluation roadmap: (i) Phase 1 (months 1–12): multi-district replication of the pilot in at least four additional districts across two states, with larger sample sizes (target *n* = 200 per cadre), to assess external validity and adaptability of the DHCF. (ii) Phase 2 (months 6–18): longitudinal retention follow-up at 3-month and 6-month intervals post-training, using both knowledge assessments and skill demonstrations, to understand how digital competencies are maintained over time. (iii) Phase 3 (months 12–24): linkage to routine data quality indicators—specifically, completeness, timeliness, and error rates in digital health records (e.g. e-Kavach entries, HMIS submissions)—to assess whether improved competencies translate to measurable improvements in service delivery data quality. (iv) Phase 4 (months 18–30): integration of UI/UX assessment (as discussed above) to evaluate the combined impact of training and tool improvements on FLHW performance and satisfaction.

## Conclusion

Despite the rapidly expanding digital responsibilities of FLHWs, there remains a paucity of standardized DHCFs to support systematic and adequate training. The DHCF represents an initial but critical step toward institutionalizing and standardizing DHCB, while providing a structured pathway to prepare FLHW cadres to function effectively within India’s ongoing digital health transformation. Using a rigorous, multi-step design process, this feasibility study developed the DHCF and demonstrated its feasibility, relevance, and practical applicability through pilot implementation and baseline assessment. The ASHA baseline assessment further validated the framework’s diagnostic utility, revealing a systematic competency hierarchy (C1L1 > C1L2 > C2L1) that directly informs the sequencing and content of cadre-specific training programs. Future research should examine the framework’s impact on key outcomes, particularly the acquisition, application, and retention of digital health competencies among FLHWs, as well as its effects on service delivery and system performance. Parallel attention to digital tool design and usability is essential to complement competency-building efforts, as training alone cannot overcome systemic tool design barriers. Given the rapidly evolving nature of digital health technologies and policies, the DHCF will require periodic review and iterative refinement to ensure continued alignment with emerging tools, standards, and workforce needs.

## Supplementary Material

Revised_Supplementary_Material_oqag009

## Data Availability

The data underlying this article (complete training content (DHCB package), the full 250-item MCQ bank, and underlying pilot data will be shared on reasonable request to the corresponding author.
