# Supplementary material for: Strengthening digital competencies in India’s health workforce: development and feasibility evaluation of a digital health competency framework for frontline healthcare workers in Uttar Pradesh
Source: Oxf Open Digit Health. 2026 May 8;4:oqag009. doi: 10.1093/oodh/oqag009 (PMC13245931; doi:10.1093/oodh/oqag009)
Supplement: Revised_Supplementary_Material_oqag009 [file revised_supplementary_material_oqag009.docx]

**Supplementary Material 1: Complete Competency dictionary**

Table S1.1-The full core competency dictionary

| **Competency Type** | **ID** | **Competency Label** | **Competency Description** | **Level 1** | **Level 2** | **Level 3** | **Level 4** | **Level 5** |
| --- | --- | --- | --- | --- | --- | --- | --- | --- |
| Digital Functional Competencies | C1 | Using digital devices and applications | Understands and uses digital devices, applications and systems for delivering the mandate efficiently | Uses basic functions of digital devices and understands its importance | Uses applications on digital devices | Proficiently uses a wide variety of technology | — | — |
|  | C2 | Data collection and management | Data collection using digital tools and management of the digital data collection process | Applies basics of digital data collection and understands its importance | Implements Data collection process | Manages and monitors data collection process | — | — |
|  | C3 | Data analysis and communication | Synthesize, analyze and communicate information for decision making | Reads basic data and understands its importance in simple decision making | Applies basics of data analysis methods and tools | Applies data analysis on a variety of data | Visualizes data and communicates using analyzed data | Decision making using data at scale |
|  | C4 | Teaching, learning and self-development | Uses digital technology for self-development and professional growth | Has knowledge of digital technologies for self-development and understands the benefits | Uses digital technology for self-development | Coaches and mentors on online learning | Creates online learning environments | — |
|  | C5 | Ethics, legal and regulatory considerations | Understands data privacy and practices consent taking | Understands data privacy and practices consent taking | Abides by legal requirements and directives in data processing | Implements data ethics policies and programs | Designs data ethics policies and guidelines | — |
| Digital Behavioral Competencies | C6 | Digital Leadership and management | Knowledge required to facilitate digital transformation | Understands digital enablers and the challenges in implementation | Promotes the use of digital technologies and builds capacity | Strengthens system capacity | Influences digital transformation trajectory | — |
| Digital Technical Domain Competencies | C7 | Introduction to Digital Health | Understands the scope and present scenario of digital health | Understands what is digital health | Understands digital health in India | — | — | — |
|  | C8 | Digital Health Informatics | Understands building blocks like EHR and interoperability | Understands, creates and utilizes EHRs for improving patient care | Understands interoperability and its utility in the healthcare ecosystem | Performs analytics for decision making | — | — |
|  | C9 | Telehealth | Utilizes remote care, telemedicine and IoT devices | Understands basics of telehealth | Implements tele health: IOT devices | Implements tele health: Tele medicine | Evaluates the impact of tele health | — |
|  | C10 | Artificial intelligence & Machine Learning | Understands, evaluates and implements AI systems | Understands the basic principles of AI & ML in healthcare | Understands the benefits and risks of AI | Uses AI systems for basic decision making | Examines bias in AI systems | Evaluates AI systems |

Table S1.2 Learning outcomes mapped to different levels of competencies

| **Competency-Level ID** | **Competency Level Label** | **Level Description 1** | **Level Description 2** | **Level Description 3** | **Level Description 4** |
| --- | --- | --- | --- | --- | --- |
| C1L1 | Uses basic functions of digital devices and understands its importance | Is aware of commonly used digital devices and accessories | Understands the need to use digital devices | Recognizes icons and understands their functions on digital devices | Is well versed in using basic functionalities (turning on, GPS, Bluetooth) |
| C1L2 | Uses applications on digital devices | Is able to install/uninstall applications and understand offline/online use | Uses commonly used apps on digital devices | Is able to create, protect and use password for logging into apps | — |
| C1L3 | Proficiently uses a wide variety of technology | Uses emerging technologies, software’s and applications | Diagnoses and resolves technical challenges and issues | Understands interoperability and performs specialist tasks across devices | — |
| C2L1 | Applies basics of digital data collection and understands its importance | Is aware of various data collection formats relevant to digital devices | Distinguishes different data collection functionalities (text, numbers, radio, dropdown) | Understands the importance of data collection using digital devices | — |
| C2L2 | Implements Data collection process | Collects evidence (images, geo-coordinates) to ensure trustworthiness | Checks for accuracy of data through a range of checks and actions | Accesses recorded data and retrieves from system/applications | — |
| C2L3 | Manages and monitors data collection process | Understands the types of errors and consistency requirements | Uses a wide range of devices to monitor and guide teams | Cleans data by focusing on error detection and missing responses | — |
| C3L1 | Reads basic data and understands its importance in simple decision making | Understands the importance of reading data in decision-making | Reads basic data and uses in simple decision making | — | — |
| C3L2 | Applies basics of data analysis methods and tools | Knows different data analysis platforms, softwares, dashboards, and Excel | Interprets and summarises data from dashboards | Understands the importance of analysing aggregated and unitized data | — |
| C3L3 | Applies data analysis on a variety of data | Knows data analysis techniques and terminologies | Analyses data through interpreting visual representation (graphs, pie charts) | Is able to operate with health information exchange | Reviews and cleans data using digital applications |
| C3L4 | Visualizes data and communicates using analyzed data | Documents and shares data in required formats (excel, word, graphs) | Identifies suitable methods of visualizing for different data types | Simplifies complex data sets into easy to read inferences | Interprets information in specialized visualizations (time series) |
| C3L5 | Decision making using data at scale | Recognises limitations of data collected and possible biases | Connects insights with context for decision making | Provides leadership for ensuring accuracy and reliability | — |
| C4L1 | Has knowledge of digital technologies for self-development | Is aware of various digital tools and apps for self-development | Understands that different tools achieve different learning levels | Understands the benefits of learning digitally | — |
| C4L2 | Uses digital technology for self-development | Is aware of and accesses online learning activities without assistance | Actively becomes a part of online learning communities | Actively seeks opportunities to learn online | — |
| C4L3 | Coaches and mentors on online learning | Mentors learners on the online learning methods suited for them | Manages and monitors professional development of others | Understands importance of coaching and mentoring | Reviews learnings and identifies new areas of development |
| C4L4 | Creates online learning environments | Learns and creates online/digital resources that support learning | Champions development of autonomous learning in others | Promotes use of wide range of innovative digital resources | — |
| C5L1 | Understands data privacy and practices consent taking | Understands concepts related to data (public, personal, confidential) | Understands data ownership and privacy | Understands consent and follows appropriate steps for taking it | — |
| C5L2 | Abides by legal requirements and directives in data processing | Is aware of legal requirements in collection, sharing, and use | Recognises and acts upon situations that might compromise data | Recognizes risks/threats of data privacy and security lapses | — |
| C5L3 | Implements data ethics policies and programs | Applies relevant laws, regulations and policies | Formulates data safety measures (anonymisation, masking, etc.) | Investigates ways of data/identity leaks and breaches | — |
| C5L4 | Designs data ethics policies and guidelines | Assesses and audits data ethics policies and practices | Monitors implementation of data ethics and practices | Drafts amendments to guidelines/policies/consent declarations | Drafts privacy and security guidelines for the organization |
| C6L1 | Understands digital enablers and challenges | Distinguishes purpose and features of various digital enablers | Understands challenges in digital implementation | Is aware of accessibility issues among different populations | Mitigates widening inequalities from introducing digital tech |
| C6L2 | Promotes digital technologies and builds capacity | Promotes confidence for professionals regarding digital use | Leads others to support technology development/national agendas | Investigates socio-technical factors impacting adoption | Aware of potential environmental implications of transformation |
| C6L3 | Strengthens system capacity | Builds relationships and collaboration with key stakeholders | Develops strategies and programmes for building capacity | Develops local standards for use of digital technologies | — |
| C6L4 | Influences digital transformation trajectory | Communicates defined vision for digital transformation | Motivates and drives mindset shift among leadership | Identifies needs of health institutions to inform strategy | Develops vision for new and futuristic transformation |
| C7L1 | Understands what is digital health | Knowledge & understanding of the term digital health | Understands various technologies and applications | Is aware of challenges (accessibility, capacity, implementation) | Understands potential benefits for seekers and providers |
| C7L2 | Understands digital health in India | Knows about various digital health schemes in India | Understands components of ABDM (ABHA, HPR, HFR, EHR) | Understands importance of ABDM adoption and associated benefits | — |
| C8L1 | Understands, creates and utilizes EHRs | Knowledge & understanding of the term EHR | Knows systems on which EHR can be created | Understands relevance of EHR in improving patient care | Aware of data quality, privacy & consent related to EHRs |
| C8L2 | Understands interoperability | Understands concept of healthcare data interoperability | Recognizes importance of interoperability for care coordination | Understands utility through everyday use-cases | — |
| C8L3 | Performs analytics for decision making | Understands importance of analytics in public health | Interprets/analyzes data to inform preparedness | Interprets data to monitor disease | Interprets data to evaluate program effectiveness |
| C9L1 | Understands basics of telehealth | Knowledge of different types of telehealth | Understands purpose and relevance using secure platforms | Aware of different digital tools and wearable devices | Understands limitations and challenges of usage |
| C9L2 | Implements tele health: IOT devices | Is aware of different wearable IoT devices | Understands use of IoT in supplementing care provision | Understands challenges associated with telehealth through IoT | Aware of best practices concerning IoT devices |
| C9L3 | Implements tele health: Tele medicine | Is aware of different tele conferencing platforms | Online patient booking, history taking, and record retrieval | Reads data and reports online and communicates to patient | Aware of telemedicine practice guidelines |
| C9L4 | Evaluates the impact of tele health | Evaluates the impact of remote care | Identifies errors and raises concern during consultation | Identifies requirements, resources, and roles for implementation | Addresses issues of consent, identity, and data security |
| C10L1 | Understands principles of AI & ML | Defines AI as tech performing tasks requiring human intelligence | Is aware of the AI technologies used in healthcare | — | — |
| C10L2 | Understands benefits and risks of AI | Identifies contribution AI makes / could make to healthcare | Articulates risks and limitations relevant to specific areas | — | — |
| C10L3 | Uses AI systems for basic decision making | Uses AI for assistance in simple decisions (e.g. drug forecast) | Knows how to respond if an AI system fails or is inaccessible | — | — |
| C10L4 | Examines bias in AI systems | Understands different types of biases (data, algorithmic, user) | Mitigates bias via inclusive design (human centred approaches) | Training with representative data and testing for bias | — |
| C10L5 | Evaluates AI systems | Compares performance against expected standards | Communicates benefits (consistency) and challenges (explainability) | — | — |

***This is used for conducting Learning outcome-based assessments tools like questions repository for written assessments,*

**Supplementary Material 2**

Table-S2: Representative MCQ items

| **C#L#** | **Question no.** | **Knowledge / Skill / Attitude** | **Question** | **Alternative A** | **Alternative B** | **Alternative C** | **Alternative D** |
| --- | --- | --- | --- | --- | --- | --- | --- |
| C1L1 | 1 | Knowledge | What is a smartphone? | It is a phone that can be used to make voice calls, video calls, access the internet, send voice notes, click and share pictures, use applications and much more. | It is a phone that is fixed to a place and can not be carried. | It is a phone that can be used only to send text messages and nothing else. | It is a phone that can be used only for making calls and sending text messages. |
|  | 2 | Knowledge | What is a feature phone? | It is a phone that can be used to make voice calls, video calls, access the internet, click and share pictures and much more. | It is a phone that has the same features as a smartphone. | It is a phone that is fixed to a place and can not be carried. | It is a phone which can be used to make calls, send messages, play music and play games. It does not have advanced features like doing video calls, etc. |
|  | 3 | Knowledge | Which of these are digital devices? | Mobile phone and tablet | Notebook and pencil | Paper and pen | Table and chair |
|  | 4 | Knowledge | For which of the following activities do you require internet active on your smartphone? | Clicking picture | Increase the volume of the phone | Making a phone call | Using Whatsapp |

| C1L2 | 1 | Skill | How do you install / download most applications? | Click on the bluetooth icon, type the name of the application, click install, after completion open app | Click on Gmail, type the name of the app, search the app in the emails, click install, after completion download the app | Click on play store, type the name of the application, click install, after completion open app | Click on settings, type the name of the application, click install, after completion open app |
| --- | --- | --- | --- | --- | --- | --- | --- |
|  | 2 | Knowledge | Do you require active internet connection for installing / downloading any app? | No | Yes |  |  |
|  | 3 | Knowledge | Do you require active internet connection for uninstalling / deleting any app? | No | Yes |  |  |
|  | 4 | Skill | How can you delete / uninstall an app you have downloaded from Play Store? | Long press the icon of the app, click on "uninstall" | Remove the app from the home screen | Click on play store, type the name of the application, click install, after completion open app | Click on settings, type the name of the application, click install, after completion open app |

| C2L1 | 1 | Knowledge | What is a data collection form? | A dashboard with data | A graph with different data points | A list of questions for which data needs to be collected | A summary table with insights from collected data |
| --- | --- | --- | --- | --- | --- | --- | --- |
|  | 2 | Knowledge | In which of the following formats can data be uploaded on digital devices? | Audio and video | Pictures | Text | All of the above |
|  | 3 | Knowledge | Which of the following is an example of data collected by uploading files? | Uploading medical records | Entering someone's age | Entering someone's date of birth |  |
|  | 4 | Knowledge | What are the different formats to collect data? | Google forms | Checklists | Taking screenshots and sharing | All of the above |

| C2L2 | 1 | Knowledge | What do you understand by "accurate data"? | The data is error-free and based on a reliable source of information | The data is full of errors | Data with some occasional errors |  |
| --- | --- | --- | --- | --- | --- | --- | --- |
| C2L2 | 2 | Knowledge | Why is it important to enter accurate data in digital devices? | Accurate data helps in better decision making for the citizens | Accurate data helps in collecting more data | Accurate data is not important at all | Accurate data is the same as inaccurate data |
| C2L2 | 3 | Knowledge | What do you understand by good quality data? | Data noted in beautiful handwriting | Data that is accurate and complete | Data that is noted in digital devices | Data that is noted in manual registers |
| C2L2 | 4 | Knowledge | How will you find out if data quality is bad? | Many columns are empty | There is a repetition of the same value many times | There is irrelevant information entered into the app/form | All of the above |

**Supplementary Material 3**

Complete observation checklist (Assessment tool for Skills)

**S 3.1**

**Competency code: C1L1, C1L2**

**Checklist**

नाम: __________________________________

उम्र: ___________________________________

अनुभव (सालों में): __________________________

पोस्टिंग कार्यालय: __________________________

**Observer Name -**

**Date and time of Observation-**

**Instructions for the examinee**- *You will be given a switched off Smartphone. You need to download the zoom application. You need to join a zoom meeting using the link shared on whatsapp. Finally delete the zoom app from the mobile phone.*

*आपको एक स्विच्ड ऑफ स्मार्टफोन दिया जाएगा। आपको ज़ूम एप्लिकेशन डाउनलोड करना होगा। आपको whatsapp पर आपके साथ शेयर्ड मीटिंग लिंक के जरिये ज़ूम मीटिंग में शामिल होना होगा। अंत में मोबाइल फोन से जूम ऐप को डिलीट कर दें।*

| **Step** | **Description** | **Yes** | **No** | **Remark** |
| --- | --- | --- | --- | --- |
| 1 | Switching on a Smartphone |  |  |  |
| 2 | Unlocking the Screen using pattern lock/ number key/ lock/ |  |  |  |
| 3 | Check for internet connection on the mobile phone  (Select Yes if this step is done even after step 6) |  |  |  |
| 4 | Connect with the internet using mobile data  (Select Yes if this step is done even after step 6) |  |  |  |
| 5 | Go to the google play store |  |  |  |
| 6 | Install zoom application from the playstore |  |  |  |
| 7 | Join meeting through the link provided on their whatsapp |  |  |  |
| 8 | Leave meeting |  |  |  |
| 9 | Uninstall the application |  |  |  |
|  | **Total** |  |  |  |

Final outcomes achieved - Yes / No

Observer signature -

**S 3.2**

**Competency code: C2L1**

**Checklist 1**

नाम: __________________________________

उम्र: ___________________________________

अनुभव (सालों में): __________________________

पोस्टिंग कार्यालय: __________________________

**Observer Name -**

**Date and time of Observation-**

Instructions for the examinee:

Exercise- A Google Form will be shared with you on WhatsApp or email. You are supposed to open the form, fill it up for all the questions, and successfully submit it.

अभ्यास-आपके साथ व्हाट्सप्प या इ-मेल पर एक गूगल फॉर्म शेयर किया जायेगा। आपका उद्देश्य है की उस फॉर्म को खोलें, उसके प्रश्नों की सूचि के उत्तर डालें, और उसे सफलतापूर्वक सबमिट करें।

| **Step** | **Description** | **Yes** | **No** | **Remark** |
| --- | --- | --- | --- | --- |
| 1 | Unlocks the phone by entering the password |  |  |  |
| 2 | Navigates to WhatsApp to open the form through the link |  |  |  |
| 3 | Opens the form by clicking on the link |  |  |  |
| 4 | Checks whether the phone is connected to the internet; connects if it isn’t |  |  |  |
| 5 | Enters data using the on-screen keyboard for Q1 and Q2 |  |  |  |
| 6 | Navigates to the correct year, month and date for entering the date of birth for Q3 |  |  |  |
| 7 | Clicks on the appropriate radio button for Q4 |  |  |  |
| 8 | Navigates to the gallery to upload a video for Q5 |  |  |  |
| 9 | Realises the square checkboxes in Q6 indicate multiple correct options  Eg: by reading all options aloud, and not stopping at the first ‘yes’ |  |  |  |
| 11 | Checks all applicable options |  |  |  |
| 12 | Clicks on the dropdown to access the options for Q7 |  |  |  |
| 13 | Selects the correct option from the dropdown in Q7 |  |  |  |
| 14 | Presses the submit button to finish filling the form and submit the data |  |  |  |
|  | **Total** |  |  |  |

Final outcomes achieved - Yes / No

Observer signature -

**Supplementary Material 4- The role-to-competency mapping tables**

Table S 3.1 - ANM Role and Activities listed

| **Responsibility** | **Activities** |
| --- | --- |
| **1. Maternal and Child Health** | 1.1 Register and provide care to pregnant women throughout the period of pregnancy. Registration of a pregnant woman for ANC should take place as soon as the pregnancy is suspected, ideally in the first tri-master (before or at the 12th week of pregnancy). However, even if a woman comes late in her pregnancy for registration, she should be registered, and care given to her according to gestational  1.2 Ensure that every pregnant woman makes at least 3 (three) visits for Ante Natal Check-up. First visit to the antenatal clinic as soon as pregnancy is suspected / between the 4th and 6th month (before 26 weeks), 2nd visit at 8th month (around 32 weeks) and 3rd visit at 9th month (around 36 weeks). Provide ante natal check ups and associated services such as IFA tablets, TT immunization etc.  1.3Test urine of pregnant women for albumin and sugar. Estimate haemoglobin level.  1.4 Refer all pregnant women to PHC for RPR test for syphilis.  1.5 Refer cases of abnormal pregnancy and cases with medical and gynaecological problems to the Health Assistant Female (LHV) or the Primary Health Centre.  1.6 Conduct deliveries in her area when called for.  1.7 Supervise deliveries conducted by Dais and assist them whenever called in.  1.8 Refer cases of difficult labour and newborns with abnormalities, help them to get institutional care and provide follow up to the patients referred to or discharged from hospital.  1.9 ANM will identify the ultimate beneficiaries, complete necessary formalities and obtain necessary approvals of the competent authority before disbursement to the beneficiaries under Janani Suraksha Yojana (JSY) and by 7th of each month will submit accounts of the previous month in the prescribed format to be designed by the State. ANM will prepare a monthly work schedule in the meeting of all accredited workers to be held on every 3rd Friday of every month, which is mandatory. The guideline under JSY is to be followed.  1.10 Make at least two post-natal visits for each delivery in her area and render advice regarding care of the mother and care and feeding of the newborn.  1.11 Assess the growth and development of the infant and take necessary action required to rectify  1.12 Educate mothers individually and in groups in better family health including maternal and child health, family planning, nutrition, immunization, control of communicable diseases, personal and environmental hygiene.  1.13 Assist Medical Officer and Health Assistant Female in conducting antenatal and postnatal clinics at the sub-centre. |
| **2. Family Planning** | 2.1 Utilise the information from the eligible couple and child register for the family Planning programme. She will be squarely responsible for maintaining eligible couple registers and updating at all times.  2.2 Spread the message of family planning to the couples and motivate them for family planning individually and in groups.  2.2 Distribute conventional contraceptives and oral contraceptives to the couples, provide facilities and to help prospective acceptors in getting family planning services, if necessary, by accompanying them or arranging for the Dai/ASHA to accompany them to hospital.  2.4 Provide follow-up services to female family planning acceptors, identify side effects, give treatment on the spot for side effects and minor complaints and refer those cases that need attention by the physician to the PHC/  2.5 Establish female depot holders, help the Health Assistant Female in training them, and provide a continuous supply of conventional contraceptives to the depot holders.  2.6 Build rapport with acceptors, village leaders, ASHA, Dais and others and utilize them for promoting Family Welfare Programme.  2.7 Identify women leaders and help the Health Assistant Female to train them.  2.8 Participate in Mahila Mandal meetings and utilize such gatherings for educating women in Family Welfare Programme. |
| **3. Medical Termination of Pregnancy** | 3.1 Identify the women requiring help for medical termination of pregnancy and refer them to the nearest approved institution.  3.2 Educate the community of the consequences of septic abortion and inform them about the availability of services for medical termination of pregnancy. |
| **4. Nutrition** | 4.1 Identify cases of malnutrition among infants and young children (zero to five years) , give the necessary treatment and advice and refer serious cases to the Primary Health Centre.  4.2 Distribute Iron and Folic Acid tablets as prescribed to pregnant nursing mothers, and young children (up to five years) as per the guidelines  4.1 Administer Vitamin A solution to children as per the  4.2 Educate the community about nutritious diets for mothers and children.  4.5 Coordinate with Anganwadi Workers. |
| **5. Universal Programme on Immunization (UIP)** | 5.1 Immunize pregnant women with tetanus toxoid.  5.2 Administer DPT vaccine, oral poliomyelitis vaccine, measles vaccine and BCG vaccine to all infants and children, (Hepatitis-B in pilot areas) as per immunization schedule.  5.3 Ensure injection safety. |
| **6. Dai Training** | 6.1 List Dais in her area and involve them in promoting Family Welfare.  6.2 Help the Health Assistant Female / LHV in the training programme of Dais. |
| **7. Communicable Diseases** | 7.1 Notify the M.O PHC immediately about any abnormal increase in cases of diarrhoea/dysentery, fever with rigors, fever with rash, fever with jaundice or fever with unconsciousness which she comes across during her home visits, take the necessary measures to prevent their spread, and inform the Health Worker Male to enable him to take further action.  7.1 If she comes across a case of fever during her home visits she will take blood smear, administer presumptive treatment and inform Health Worker male for further action.  7.2 Identify cases of skin patches, especially if accompanied by loss of sensation, which she comes across during her home visits and bring them to the notice of the Health Worker Male/MO (PHC).  7.4 Assist the Health Worker Male in maintaining a record of cases in her area, who are under treatment for malaria, tuberculosis and leprosy, and check whether they are taking regular treatment, motivate defaulters to take regular treatment and bring these cases to the notice of the Health Worker Male or Health Assistant Male.  7.5 Give Oral Rehydration solution to all cases of diarrhea/dysentery/vomiting. Identify and refer all cases of blindness including suspected cases of cataract to M.O. PHC.  7.6 Education, Counselling, referral, follow-up of cases STI/RTI, HIV/AIDS.  7.7 Where Filaria is endemic:  ♦ Identification of cases of lymphoedema / elephantiasis and hydrocele and their referrals to PHC/CHC for appropriate management.  ♦ Training of patients with lymphoedema / elephantiasis about care of feet and with home based management remedies.  ♦ Identification and training of drug distributors for mass drug distribution of DEC on National Filaria Day. |
| **8. Vital Events** | 8.1. Record and report to the health authority of vital events including births and deaths, particularly of mothers and infants to the health authorities in her area.  8.2. Maintenance of all the relevant records concerning mother, child and eligible couples in the area. |
| **9. Record Keeping** | 9.1 Register  (a) pregnant women from three months of pregnancy onward  (b) infants zero to one year of age; and (c) women aged 15 to 44 years.  9.2 Maintain the pre-natal and maternity records and child care records.  9.3 Prepare the eligible couple and child register and maintaining it up-to-date  9.4 Maintain the records as regards contraceptive distribution, IUD insertion. Couples sterilized, clinics held at the sub-centre and supplies received and issued.  9.5 Prepare and submit the prescribed weekly / monthly reports in time to the Health Assistant Female.  9.6 While maintaining passive surveillance register for malaria cases, she will record:  ♦ No. of fever cases  ♦ No. of blood slides prepared  ♦ No. of malaria positive cases reported  ♦ No. of cases given radical treatment |
| **10. Treatment of minor ailments** | 10.1 Provide treatment for minor ailments, provide first-aid for accidents and emergencies and refer cases beyond her competence to the Primary Health Centre/Community Health Centre or nearest hospital. |
| **11 Team Activities** | 11.1 Attend and participate in staff meetings at Primary Health Centre/Community Development Block or both.  11.2 Coordinate her activities with the Health Worker Male and other health workers including the Health volunteers/ASHA and Dais.  11.3 Coordinate with the PRI and Village Health and Sanitation Committee  11.4 Meet the Health Assistant Female each week and seek her advice and guidance whenever necessary.  11.5 Maintain the cleanliness of the sub-centre.  11.6 Dispose medical waste as per the guidelines.  11.7 Participate as a member of the team in camps and campaigns. |

##

## Table S3.2 ANM FRAC Role, Activities & Competency Mapping

| **Role** | **Key Activities (with Digital Health Aspects)** | **Mapped Competency Levels** |
| --- | --- | --- |
| **Maternal and Child Health** | Register PW in 1st trimester; Track ANC visits (3+); Test urine/Hb; HRP identification and referral; JSY approvals and monthly work schedules using digital health records and registration forms. | C1L1, C2L3, C3L2, C7L2 |
| **Family Planning** | Maintain/update Eligible Couple Register; Motivation and distribution of contraceptives; Establish depot holders; Identify women leaders using digital registers and inventory tracking. | C1L1, C2L2, C7L2 |
| **Medical Termination of Pregnancy** | Identify and refer women for MTP; Community education on septic abortion using digital referral platforms and educational tools. | C1L1, C2L2, C7L2 |
| **Nutrition** | Identify malnutrition (0-5 yrs); Distribute IFA/Vit A; Coordinate with AWWs using digital assessment tools and growth monitoring apps. | C1L1, C2L3, C7L2 |
| **Universal Programme on Immunization (UIP)** | Immunize PW and children (BCG, DPT, OPV, Measles, Hep-B); Ensure injection safety using digital immunization trackers (U-WIN/e-Vin). | C1L1, C2L2 |
| **Dai Training** | List and involve Dais; Help LHV/Health Assistant in training programs using digital learning materials and communication tools. | C1L1, C3L3, C5L3 |
| **Communicable Diseases** | Notify MO on outbreaks; Take blood smears; Identify Leprosy patches; ORS/Blindness referral; STI/HIV follow-up; Filaria tracking using digital surveillance (IDSP) and reporting tools. | C1L1, C2L4, C5L2, C7L2 |
| **Vital Events** | Record and report births and deaths; Maintain mother/child and eligible couple records using digital civil registration systems (CRS) and RCH portals. | C1L1, C2L2, C5L2, C7L2 |
| **Record Keeping** | Register PW, infants, and women (15-44 yrs); Maintain maternity/contraceptive records; Weekly/monthly reporting using electronic medical records and data entry apps. | C1L1, C2L2, C7L2 |
| **Treatment of Minor Ailments** | Provide first-aid and emergency care; Refer cases beyond competence using digital diagnostic aids and referral platforms. | C1L1, C2L2, C7L2 |
| **Team Activities** | Participate in PHC meetings; Coordinate with ASHA, Dais, and PRI; Dispose medical waste; Participate in campaigns using digital communication and scheduling tools. | C1L1, C3L3 C5L2 |

## Table S3.3 ASHA FRAC Role, Activities & Competency Mapping

| **Role** | **Key Activities & Tasks** | **Competency Levels** |
| --- | --- | --- |
| **Making of Village Health Plan** | It is a framework for health-related action | C1L1, C1L2 |
| **Communication for Health Behavior Change** | Interpersonal communication for driving health behavior change | C1L2 |
| **Linkages with AWW, TBA, ANM, MPW** | Coordinating between different health workers and sectors | C2L1 |
| **Counseling** | Providing guidance on health, nutrition, and hygiene | C4L1; C5L2 |
| **Escorting patients to a hospital** | Accompanying pregnant women or cases requiring higher care | C5L1 |
| **Primary Medical Care** | Providing first-aid and basic medical assistance | C4L2 |
| **Act as Depot Holders** | Maintaining stocks of essential health kits/medicines | C7L1 |
| **Records and Registrations** | Register every birth/still birth (14 days); Register every death (7 days); Keep record in ASHA register/diary; Note experiences/difficulties; Tally records with Anganwadi/Sub-center | C1L2 |

## S3.4 Anganwadi worker (AWW): Mapping of Role, Activities & competency

| **Role** | **Key Activities & Tasks** | **Mapped Competency Levels** |
| --- | --- | --- |
| **Nutrition and Health Education** | Prepare nutrition corners; Organize Godbharai/Annaprashan; Demonstrate diet diversity and breastfeeding/complementary feeding practices. | C1L4 |
| **Pre-School Non-Formal Education** | Mobilize children (3-6 years); Carry out ECCE activities as per yearly calendar; Conduct half-yearly child assessments and update cards. | C1L3 |
| **Monitor Growth of Children** | Measure weight and height (0-6 years); Identify undernutrition (stunting/wasting); Follow-up during home visits and growth monitoring sessions. | C2L3 |
| **Support in Immunization** | Supports Immunization of the target population as per the national immunization schedule. | C3L3 |
| **Support in Health Check-ups** | Mobilize Pregnant women and children (0-6 yrs) for regular health check-ups at available platforms (VHND, RBSK etc.). | C4L2 |
| **Supplementary Nutrition (HCM & THR)** | Update child lists; Coordinate with schools; Procure, store, and supervise HCM/THR distribution; Ensure hygiene/handwashing; Follow-up on consumption. | C7L2 |
| **Appropriate Referral Services** | Refer beneficiaries to avail appropriate health services; Discuss issues related to referral services in sector meetings. | C2L3 |
| **Record Maintenance of ICDS** | Maintain HCM/THR registers, Poshan Tracker, Growth Monitoring data, CBE records (Annaprashan/Godbharai), and referral data. | C7L2 |
| **Counseling to Mothers/Caregivers** | Counsel on THR benefits, maternal nutrition, breastfeeding (0-6m), and optimal feeding practices (6-23m). | C5L2 |

## Supplementary Material 5

## Table S4 Comparative Analysis of Digital Health Competency Frameworks

| ***Dimension/ Framework*** | **ANMF** | **NHS** | **DHCF by VDH** | **FRAC** | **DHCF** |
| --- | --- | --- | --- | --- | --- |
| **Target workforce** | Nurses and Midwives | Health Professionals, including nurses, AHPs, and doctors | Allied Health Professionals | Government Employees across sectors | Frontline Health Workers |
| **Applicability to low and middle- income settings** | Low - developed for a high-income health system | Low - designed for a highly digitised system in a high-income setting | Low - developed for a state-level public health system in a high-income setting | High - designed for government employees in low-resource settings | High - designed for FLHWs working in remote and low-resource settings |
| **Competency Assessment Approach** | Profession-  specific (nurses and midwives), domain-based | System-wide, domain-based and role-agnostic archetype mapping | Domain-based | Domain, function and behaviour - based, mapped to specific roles and activities | Domain, function and behaviour -based, mapped to specific roles and activities |
| **Proficiency structure** | 3 levels - formative, intermediate, proficient | 4 levels of knowledge, increasing in complexity and depth | 4 levels of expertise- Foundation, Consolidation, Expert, Leader | 5 levels, progressing from basic to advanced competency | 5 levels, progressing from basic to advanced competency |
| **Domains** | Digital professionalism, leadership & advocacy, data and information quality, information-  enabled care, technology | Digital implementation, digital care delivery, ethics/legal, human factors, data management, AI and emerging technologies | Digital workplace, Digital Professionalism, Data/Informatics,  Digital transformation | No fixed domains; competencies are task-derived and grouped into behavioural, functional, and domain categories. | Using digital devices and applications, Data collection and management, Data analysis and communication,  Data privacy and consent. |
| **Evaluation component** | Self-assessment and reflection | Guided self-assessment and training alignment | Structured self-assessment tools | Formal, system- integrated assessment | Formal, integrated assessments |
| **Institutional alignment** | Designed as a professional development and workforce planning tool used by employers, educators, and individuals. | Embedded in national workforce planning and digital transformation initiatives across the NHS. | Developed by a state health department to guide workforce capability, organisational benchmarking, and training programs. | Mandated and supported by central governance structures and integrated into digital platforms for continuous capacity building. | Designed for multi-level integration -  led by state and district health authorities and embedded into routine reviews, mentoring, and frontline worker platforms |

**Appendix C: Minimum Viable Blended Training Approach**

Based on the pilot findings, the following minimum viable blended training approach is

recommended for DHCF implementation:

***Phase 1*** – In-Person Introduction (1 session, approximately 60 minutes): Delivered during

existing cluster meetings or ANM weekly meetings at CHCs. Covers orientation to the DHCF,

demonstration of basic device functions, and hands-on practice with guided facilitation. Pre-

assessment administered at session start.

***Phase 2*** – Self-Paced Micro-Modules (4 modules, 15–20 minutes each): Hosted on the e-

Kshamata LMS (web-based and Android app). Modules cover: (i) Basic device functions and

importance, (ii) Applications on digital devices, (iii) Basics of digital data collection, (iv) Data

privacy and consent. Each module includes embedded knowledge checks and a short post-

module quiz.

***Phase 3*** – Monthly Reinforcement (ongoing): Activities embedded in monthly cluster meetings,

including peer-support practice sessions (15 minutes), troubleshooting discussions for common

digital challenges, and refresher quizzes. WhatsApp peer-support groups facilitate continuous

learning between meetings. Tech Mitras (block-level digital support persons) provide on-demand

technical assistance.

***Phase 4*** – Assessment and Certification: Post-assessment (knowledge MCQs) administered after

completion of all micro-modules. Skill demonstration assessment conducted during a dedicated

cluster meeting session. Certification is issued upon achieving threshold scores on both

knowledge and skill assessments. Refresher training triggered for those below the threshold.

Suggested Timeline: Weeks 1–2: In-person introduction; Weeks 3–6: Self-paced micro-modules;

Week 7 onwards: Monthly reinforcement with assessment at Week 8. This pacing allows

integration with existing training calendars without requiring additional dedicated training days.
